# Clinical Interchangeability Boundaries of Pentacam AXL WAVE in Preoperative Biometry for Cataract Surgery: An Agreement Study with IOLMaster 700 and Pentacam

**DOI:** 10.3390/jcm15176599

**Published:** 2026-08-26

**Authors:** Rong Zhao, Zhiyang Zhang, Suyu Wang, Ziyi Chen, Guangguang Wu, Siyuan Ding, Qin Jiang, Jiajun Li, Keran Li

**Affiliations:** 1 Department of Ophthalmology, Eye Hospital of Nanjing Medical University, Nanjing 210029, China; joy972@163.com (R.Z.); zzhiyang2002@163.com (Z.Z.); wang__suyu@stu.njmu.edu.cn (S.W.); chenziyi@stu.njmu.edu.cn (Z.C.); simonmeddoc@163.com (G.W.); 2024121868@stu.njmu.edu.cn (S.D.); 2 The Fourth School of Clinical Medicine, Nanjing Medical University, Nanjing 210029, China

**Keywords:** Pentacam AXL WAVE, IOLMaster 700, Pentacam, cataract surgery, ocular biometry, interdevice agreement

## Abstract

**Background/Objectives**: Accurate preoperative biometry is essential for refractive cataract surgery. Pentacam AXL WAVE is an emerging multimodal platform integrating ocular biometry, anterior segment imaging, and corneal assessment, yet its interchangeability with established devices remains unclear. This study aimed to evaluate the agreement and clinical interchangeability of Pentacam AXL WAVE with IOLMaster 700 and Pentacam. **Methods**: This retrospective cross-sectional study compared Pentacam AXL WAVE with IOLMaster 700 and Pentacam across 175 eyes of 105 cataract patients. Interdevice agreement was evaluated using intraclass correlation coefficients, concordance correlation coefficients, and Bland–Altman analysis with multiple outlier-handling strategies; generalized estimating equations were applied to identify clinical factors associated with interdevice differences. **Results**: Pentacam AXL WAVE demonstrated good agreement with IOLMaster 700 and Pentacam for axial length and anterior corneal curvature parameters, but showed weaker agreement for white-to-white diameter, total keratometry, total corneal astigmatic vectors, and several extended anterior segment parameters. Fewer clinical factors explained interdevice differences versus IOLMaster 700, whereas ocular surface or corneal abnormalities, refractive status, and cataract characteristics were associated with greater differences versus Pentacam. **Conclusions**: These findings indicate that Pentacam AXL WAVE has parameter-specific interchangeability boundaries and can meet some routine biometric needs; however, for eyes requiring high refractive precision or presenting with complex ocular conditions, interpretation should be based on the agreement characteristics of individual parameters.

## 1. Introduction

As cataract surgery has fully entered the refractive era, the focus has shifted beyond lens extraction toward optimizing postoperative visual outcomes. The increasing use of multifocal, extended depth of focus (EDOF), and toric intraocular lenses (IOLs) has heightened the importance of accurate preoperative ocular biometry in achieving refractive targets and patient satisfaction. Even small errors in measurements such as axial length (AL), keratometry (K), total keratometry (TK), and anterior chamber depth (ACD) may be magnified by IOL power formulas, leading to postoperative refractive surprises [1].

Contemporary ocular biometry devices employ a range of optical technologies, including partial coherence interferometry (PCI), swept-source optical coherence tomography (SS-OCT), and rotating Scheimpflug imaging. These devices differ in light-source wavelength, tissue penetration, imaging modality, reference axis, measurement zone, and calculation algorithms, which can introduce systematic measurement bias on certain parameters. Previous studies have shown high correlations between devices for AL and K values, but correlation alone does not imply clinical interchangeability [2,3]. This issue is more pronounced for total keratometry and total corneal astigmatic vector components. Differences in measurement zones and calculation models can be amplified [4]. It remains uncertain whether such interdevice differences during preoperative cataract assessment are within acceptable clinical limits.

In addition to device differences, clinical characteristics of cataract patients also influence measurement accuracy. The severity and morphology of lens opacity can limit the penetration of the optical signal and degrade image quality [5,6]. Variations in refractive status alter corneal morphology and refractive properties, thereby affecting accuracy [7]. Anterior or posterior segment diseases may also interfere with measurements by changing corneal morphology, tear film stability, boundary recognition, or intraocular light scatter [8,9]. Previous studies have shown that in complex cases, such as dense or posterior subcapsular cataracts, SS-OCT-based devices outperform PCI devices for AL measurement [10]. Therefore, evaluating how these clinical characteristics relate to device agreement is essential.

Pentacam AXL WAVE is a multimodal anterior segment and biometric device. By integrating Scheimpflug imaging, axial biometry, and retroillumination analysis, it enables the acquisition of AL, corneal curvature, anterior chamber parameters, and additional anterior segment metrics within a single examination. This integrated workflow may streamline preoperative assessment for cataract patients. Multiple functions, however, do not imply that all of their measures can take the place of those from current equipment. There is still a paucity of comprehensive, parameter-based comparisons between its measurements and those from IOLMaster 700 and Pentacam.

Based on these considerations, the study enrolled patients undergoing preoperative cataract assessment, using Pentacam AXL WAVE as the primary device of interest. It evaluated agreement and clinical interchangeability with the IOLMaster 700 and Pentacam for ocular biometric parameters, including AL, ACD, K values, TK, white-to-white distance (WTW), astigmatic vectors, and anterior segment measures. The robustness of the outcome was further evaluated through outlier analyses. In addition, the study explored factors associated with interdevice measurement differences in relation to cataract type, refractive status, and ocular disease, to help guide the clinical use of these devices and inform device selection during preoperative cataract assessment.

## 2. Materials and Methods

### 2.1. Study Design and Study Population

This was a single-center, retrospective, observational, cross-sectional study. It included patients who had preoperative cataract assessment at the Eye Hospital of Nanjing Medical University between 12 November 2025 and 11 December 2025. The study followed the principles of the Declaration of Helsinki. It was approved by the Ethics Committee of the Eye Hospital of Nanjing Medical University (approval No. 2025-LSKY-2025015). Given the nature of the study, informed consent was waived.

The study population consisted of patients scheduled for cataract surgery who underwent preoperative ocular biometry. Inclusion criteria were age > 40 years, a clinical diagnosis of age-related cataract in the study eye, and at least one paired preoperative biometric assessment obtained with Pentacam AXL WAVE and either the IOLMaster 700 or the Pentacam. To improve the representativeness of the study population in a real-world preoperative cataract setting, no additional clinical exclusion criteria were predefined. Eyes without reliable measurements or lacking paired values were excluded from the analysis for the corresponding parameters.

A total of 175 eyes from 105 patients were included in this study. For patients meeting criteria and with valid measurements in both eyes, data from both were included. Given the potential correlation between the two eyes of the same patient, statistical models accounting for within-subject clustering were used to analyze clinical factors.

In addition, since not all eyes completed measurements on all three devices, and some parameters had missing or quality-control-rejected values, the final sample size for the paired-agreement analysis varied across parameters. For each parameter, only paired data with valid measurements obtained from both devices were included in the analysis.

### 2.2. Preoperative Measurement Devices and Examination Workflow

All included eyes underwent routine preoperative ophthalmic examinations for cataract surgery and were examined with Pentacam AXL WAVE, IOLMaster 700, and/or Pentacam according to the clinical examination workflow. Only paired data with valid results from both devices under comparison and passing quality control were included in each analysis. All examinations were performed according to the manufacturers’ standard protocols by trained examiners under standardized conditions. During examination, patients were seated comfortably with the chin positioned on the chin rest and the forehead against the forehead strap. Patients were instructed to keep both eyes naturally open and fixate on the internal target. And they were asked to blink immediately before measurement to reduce the potential influence of tear-film instability. The examiner moves the joystick to focus, and the instrument automatically measures and displays the parameters. Only data meeting the instrument’s quality control standards are accepted. It should be noted that data acquisition for the Pentacam AXL Wave and Pentacam must be carried out under dim room illumination.

Pentacam AXL WAVE (OCULUS Optikgeräte GmbH, Wetzlar, Germany, software version 1.34) is based on a rotating Scheimpflug imaging platform with integrated axial biometry. It enables three-dimensional imaging of the cornea and anterior segment, yielding parameters describing the anterior and posterior corneal surfaces and the anterior chamber. Axial length is measured using intergrated eye tracking. Therefore, the device can provide both corneal morphological and ocular biometric parameters within a single examination platform. In the present study, Pentacam AXL WAVE was used as the primary study device. Its measurements was compared with IOLMaster 700, which is based on a different measurement principle, for corresponding ocular biometric parameters, and with conventional Pentacam for shared corneal parameters.

IOLMaster 700 (Carl Zeiss Meditec AG, Jena, Germany, version 1.88.1) uses swept-source optical coherence tomography (SS-OCT) to perform non-contact ocular biometry by detecting optical coherence signals from different intraocular tissue interfaces. It provides commonly used preoperative biometric parameters, including axial length, corneal curvature, anterior chamber depth, and white-to-white distance. Unlike Pentacam AXL WAVE, which combines Scheimpflug imaging with integrated axial biometry, IOLMaster 700 primarily relies on SS-OCT for ocular biometric measurements.

Pentacam (Oculus Optikgeräte GmbH, Wetzlar, Germany, version 1.27r13) uses rotating Scheimpflug imaging to reconstruct three-dimensional anterior segment structures from tomographic images acquired continuously at different angles. It provides measurements of anterior and posterior corneal surface morphology, corneal curvature, corneal thickness, and anterior chamber parameters. Compared with Pentacam AXL WAVE, conventional Pentacam is primarily used for anterior segment and corneal morphological assessment and does not include the integrated axial biometry function available in the AXL WAVE platform.

Measurement results from all devices were obtained from the automatic system outputs. Before statistical analysis, the raw data were checked for paired consistency, screened for missing values, assessed for quality control, and standardized in format. For parameters containing directional or spatial positional information, such as astigmatism and Chord α/μ, axial extraction, vector transformation, or decomposition into X/Y components was further performed according to predefined rules before inclusion in the analysis.

### 2.3. Biometric Parameters Included in the Analysis

Pentacam AXL WAVE served as the common comparison device and was compared independently with the IOLMaster 700 and Pentacam. The included parameters mainly comprised commonly used preoperative biometric indices for cataract surgery, including AL, ACD, WTW/HWTW, anterior corneal curvature parameters such as K1, K2, SE, and anterior-surface astigmatism-related indices, as well as total keratometry parameters such as TK1, TK2, TSE, and total corneal astigmatism-related indices. Parameters available across different devices and considered clinically comparable were included in the agreement analyses.

In addition, selected extended anterior segment parameters output by Pentacam and Pentacam AXL WAVE were included in exploratory analyses, including pupil diameter, Belin/Ambrósio deviation index (BAD-D), corneal optical density (COD), corneal thickness at the vertex (Pachy Vertex N), thinnest corneal thickness, axial/sagittal B/F ratio, anterior chamber angle-related parameters, Chord α, and Chord μ.

### 2.4. Astigmatic Vector Transformation and Chord Parameter Decomposition

To more accurately evaluate agreement across devices in astigmatism measurements, vector transformation analysis was performed on astigmatic magnitude and axis [11]. Anterior corneal astigmatism and total corneal astigmatism were separately transformed into vector components to reduce the influence of the periodic nature of the astigmatic axis on statistical results.

Specifically, the Jackson cross-cylinder notation was used to decompose astigmatism into two vector components, J0 and J45, where J0 represents the 0°/90° component, and J45 represents the 45°/135° component. The corresponding formulas were as follows [12]:

J0 = (−C/2) cos(2α),(1)

J45 = (−C/2) sin(2α),(2)

where C denotes the cylindrical power of astigmatism and α denotes the astigmatic axis. The anterior corneal astigmatic vector components were denoted as K_J0 and K_J45, whereas the total corneal astigmatic vector components were denoted as TK_J0 and TK_J45.

For Chord α and Chord μ, to more comprehensively characterize their spatial positional information, this study retained the radial distance parameter (r) and further decomposed each parameter into two orthogonal components, X and Y, for analysis [13]. The X component indicates horizontal displacement, the Y component indicates vertical displacement, and r represents the radial distance of the displacement. For devices that provided the corresponding outputs, only clinically comparable Chord α and Chord μ parameters were included, and paired comparisons and agreement analyses were performed based on their X and Y components and radial distance (r) [14].

### 2.5. Outlier Definition and Sensitivity Analysis

To evaluate the influence of abnormal interdevice differences on agreement results, outlier identification strategies were predefined in this study. For routine biometric parameters with clear clinical interpretability, the clinical thresholds for the primary analysis were determined based on the previous literature, clinically acceptable error ranges, and the predefined criteria of this study. An outlier was defined as an absolute difference between the two device measurements that reached or exceeded the corresponding predefined threshold, as follows:

|Device A − Device B| ≥ predefined threshold.(3)

In this study, the clinical thresholds for the primary analysis were set at 0.10 mm for AL [15,16], 0.10 mm for ACD [17,18], and 0.50 mm for WTW/HWTW [19]. For refractive power-related parameters, including K1, K2, SE, TK1, TK2, and TSE, the primary clinical threshold was set at 0.50 D, and a stricter threshold of 0.25 D was further applied in the sensitivity analyses [20,21,22]. For astigmatic magnitude and astigmatic vector components, including K_J0, K_J45, TK_J0, and TK_J45, the primary clinical threshold was set at 0.25 D, with 0.125 D used as the stricter threshold in the sensitivity analyses [21]. For extended anterior segment parameters without established clinical thresholds, including ACA180, BAD-D, COD, pupil diameter, thinnest corneal thickness, and the X/Y components of Chord α/μ, distribution-based outlier detection methods were used instead of prespecified clinical-threshold criteria, as shown in Table 1.

In addition to the clinical-threshold method, two distribution-based outlier detection methods were applied as sensitivity analyses. For the Tukey 1.5 × interquartile range (IQR) method [23], the interquartile range of the absolute difference (abs_diff) was calculated, and observations were classified as IQR outliers if they met the following criterion:

abs_diff > Q3 + 1.5 × IQR.(4)

For the median absolute deviation (MAD) method [24,25], a modified z score was similarly calculated based on abs_diff, and observations were classified as MAD outliers if they met the following criterion:

|modified z score| > 3.5(5)

The 95% limits of agreement (LoA) were used only to describe the distribution range of interdevice differences and were not used as outcome variables in the regression models.

### 2.6. Statistical Analysis

All statistical analyses were performed using R (version 4.5.1). Continuous variables were expressed as mean ± standard deviation or median with interquartile range, depending on their distribution, whereas categorical variables were expressed as counts and percentages.

Sample-size adequacy and statistical power were assessed using an intraclass correlation coefficient (ICC)-based approach [26]. A minimum acceptable ICC of 0.75 and an expected ICC of 0.90 were specified, with a two-sided α of 0.05, two device measurements per comparison, and a target power of 90%. Under these assumptions, the minimum required sample size was 44 independent participants. Because some participants contributed measurements from both eyes, the patient count rather than the eye count was used for this assessment to avoid overestimating the effective sample size. The study included 105 patients, exceeding the minimum required sample size, with a corresponding statistical power of 99.91%.

For each device, paired screening was first performed, and only eyes with valid measurements from both devices were included in the analysis of the corresponding parameter.

Differences in the same parameter between two devices were compared using the paired *t*-test or the Wilcoxon signed-rank test, depending on the data distribution. Interdevice agreement was evaluated using Bland–Altman analysis [27], ICC [28], and concordance correlation coefficient (CCC) [29]. Bland–Altman analysis was used to estimate the mean bias and 95% LoA, and to graphically display the distribution of interdevice differences. For parameters with proportional bias or heteroscedasticity, logarithmic transformation or regression-based Bland–Altman methods were used for supplementary analyses [30]. ICC was estimated using a two-way mixed-effects, absolute-agreement, single-measurement model [31], whereas CCC was calculated according to Lin’s method [29]. The primary agreement analysis was first performed in the full sample and then repeated after excluding predefined clinical-threshold outliers, strict clinical-threshold outliers, IQR outliers, and MAD outliers to assess the robustness of the results.

To further explore the relationship between clinical characteristics and interdevice measurement differences, multivariable regression models were constructed using interdevice difference indices as outcome variables. Considering the potential correlation between fellow-eye data from the same patient, all eye-level regression analyses were performed using generalized estimating equation (GEE) [32,33], with an exchangeable working correlation structure and robust standard errors to account for within-subject clustering.

Different forms of outcome variables were used for modeling based on clinical relevance, outlier rates, and distributional characteristics of the differences for each parameter. For parameters with clearly defined clinical inconsistency thresholds and a sufficient number of outlier events, whether the predefined threshold was reached was used as a binary outcome, and logistic GEE analysis was performed. For parameters with low outlier rates but clinically interpretable difference magnitudes, the absolute difference was treated as a continuous outcome. When the distribution of absolute differences was markedly skewed, analyses were conducted following log (abs_diff + offset) transformation [34]. For parameters with evident directional systematic bias, or for which the direction of the difference was clinically interpretable, the directional difference (device A − device B) was used as a continuous outcome. All continuous outcomes were analyzed using Gaussian GEE.

A unified covariate framework was used for the analyses of clinical factor associations. Candidate covariates were predefined based on clinical relevance, including age, laterality, refractive status, cataract type, Lens Opacities Classification System III (LOCS III) grading, and ocular comorbidity variables. Refractive status was further divided into two indicators, degree of myopia and degree of hyperopia. Ocular comorbidities were categorized according to anatomical and clinical relevance into ocular surface, anterior segment, and posterior segment disease variables. To reduce the risk of overfitting, variable inclusion in each model was controlled based on sample size, the number of outlier events, variable completeness, and model convergence.

## 3. Results

### 3.1. Subsection Demographic and Clinical Characteristics

A total of 175 eyes from 105 patients undergoing preoperative cataract assessment were included, comprising 31 men (29.5%) and 74 women (70.5%). Among the included eyes, 86 (49.1%) were right eyes and 89 (50.9%) were left eyes. The mean age of the participants was 67.72 ± 9.28 years. Two interdevice comparisons were evaluated: Pentacam AXL WAVE versus IOLMaster 700 (Group 1) and Pentacam AXL WAVE versus Pentacam (Group 2). For each comparison, agreement was assessed in the full sample and after applying different outlier-handling methods. Factors associated with interdevice measurement differences were also analyzed.

### 3.2. Primary Agreement Analysis

**Figure 1:**
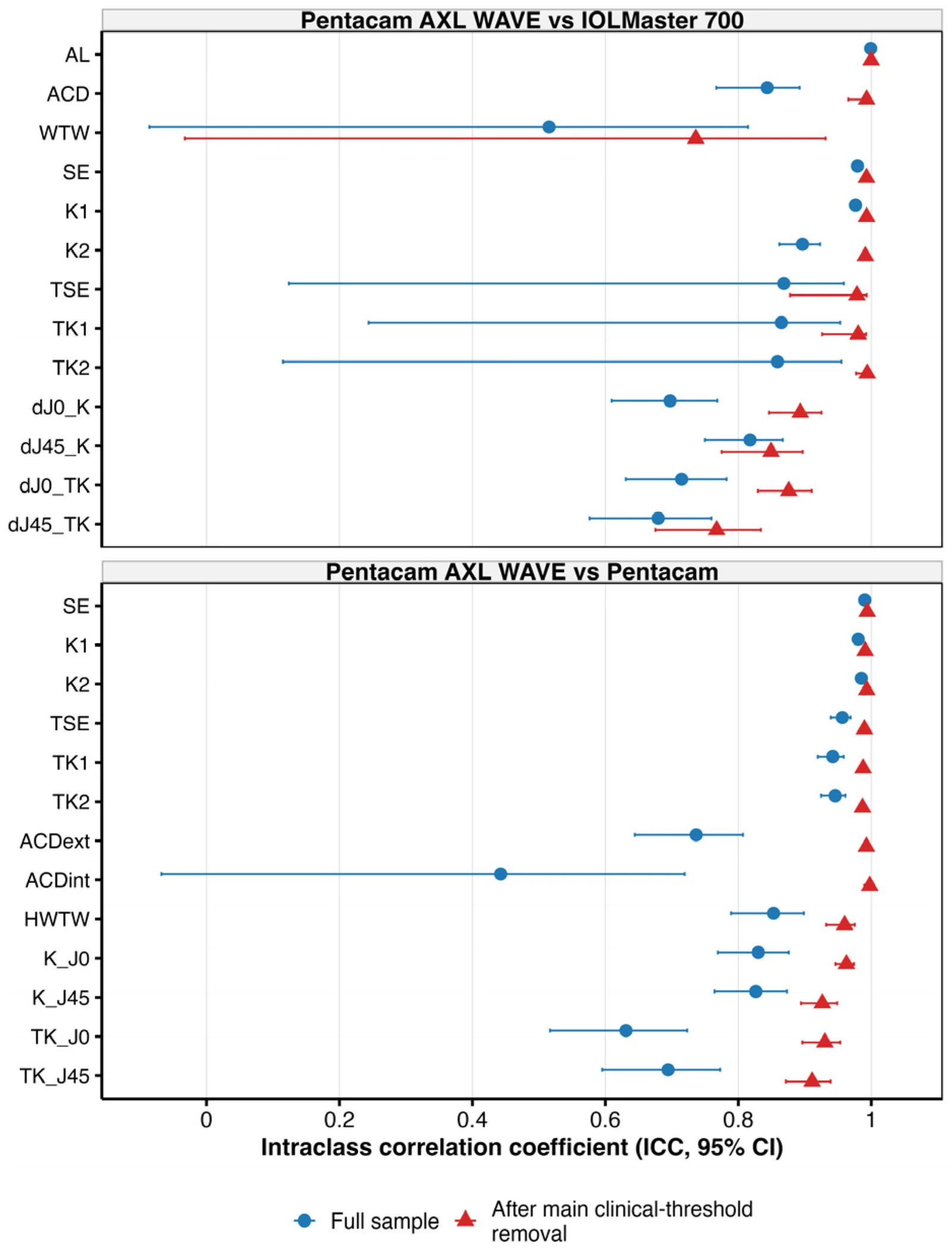
ICC forest plot for major biometric parameters. The forest plot shows the ICC and 95% confidence intervals for major biometric parameters and the astigmatism vector components in two comparisons: between the Pentacam AXL WAVE and the IOLMaster 700, and between the Pentacam AXL WAVE and the Pentacam. Blue dots represent the results of the full sample analysis, whilst red triangles represent the results of the analysis after excluding outliers based on the primary clinical threshold.

ICC, CCC and Bland–Altman analyses were performed separately for the full sample and for the dataset obtained after excluding primary clinical-threshold outliers. ICC results for the main parameters are presented in a forest plot to facilitate visual comparison between the two device comparisons before and after outlier exclusion (Figure 1). Detailed results for ICC, CCC, bias, and 95% LoA are provided in Supplementary Tables S1 and S2.

In the comparison between Pentacam AXL WAVE and IOLMaster 700, AL showed the most stable agreement, with ICCs approaching 1.00 in both the full sample analysis and analysis after exclusion of primary clinical-threshold outliers. ACD, SE, K1, and K2 improved markedly after eyes with large interdevice differences were excluded. In contrast, WTW continued to show only low-to-moderate agreement across different analytical conditions, suggesting that its interdevice differences were not primarily driven by a small number of outliers. TSE, TK1, and TK2 showed higher ICCs after outlier removal, but the remaining sample size was substantially reduced, indicating that good agreement was observed only in a highly selected subset (Figure 1; Supplementary Table S1).

In the comparison between Pentacam AXL WAVE and Pentacam, SE, K1, and K2 showed high agreement even in the full sample, suggesting good comparability between the two Scheimpflug-based devices for anterior corneal curvature-related parameters. ICCs for TSE, TK1, and TK2 were also generally high, although their 95% LoA should still be interpreted in relation to clinical thresholds. In contrast, extended anterior segment parameters, including internal ACD (ACDint), ACA180, COD, and Chord α/μ-related components, showed weaker agreement, suggesting limited clinical interchangeability (Figure 1; Supplementary Table S2).

Astigmatic vector analysis revealed a consistent pattern across both comparisons, whereby anterior-surface vectors components tended to demonstrate better agreement than total corneal vectors components. In Group 1, all astigmatic vector components showed improved agreement after exclusion of primary clinical-threshold outliers, but overall agreement remained weaker than that of routine corneal curvature parameters. In Group 2, K_J0 and K_J45 showed better agreement than TK_J0 and TK_J45, indicating that total corneal astigmatic vectors were more susceptible to interdevice differences than anterior-surface astigmatic vectors (Figure 1; Supplementary Tables S1 and S2).

**Figure 2:**
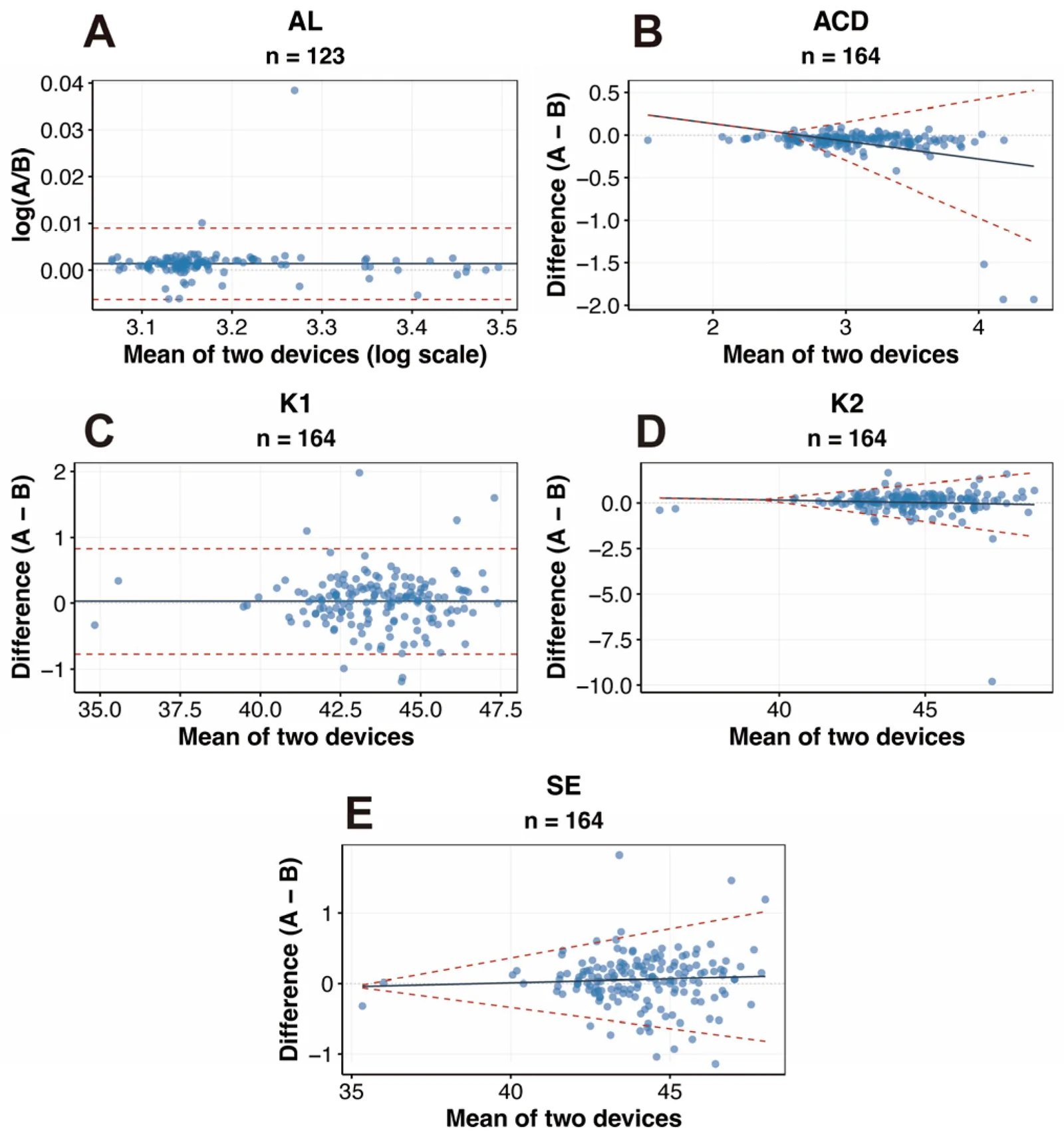
Bland–Altman analysis between AXL WAVE and IOLMaster 700. The inter-device consistency of AL (**A**), ACD (**B**), K1 (**C**), K2 (**D**) and SE (**E**) is shown. The vertical axis represents the difference between the measurements of the two devices; the horizontal axis represents the mean of the measurements from the two devices. The results for AL are presented on a logarithmic scale. The grey solid line represents the estimated bias, and the red dashed lines represent the 95% limits of agreement.

**Figure 3:**
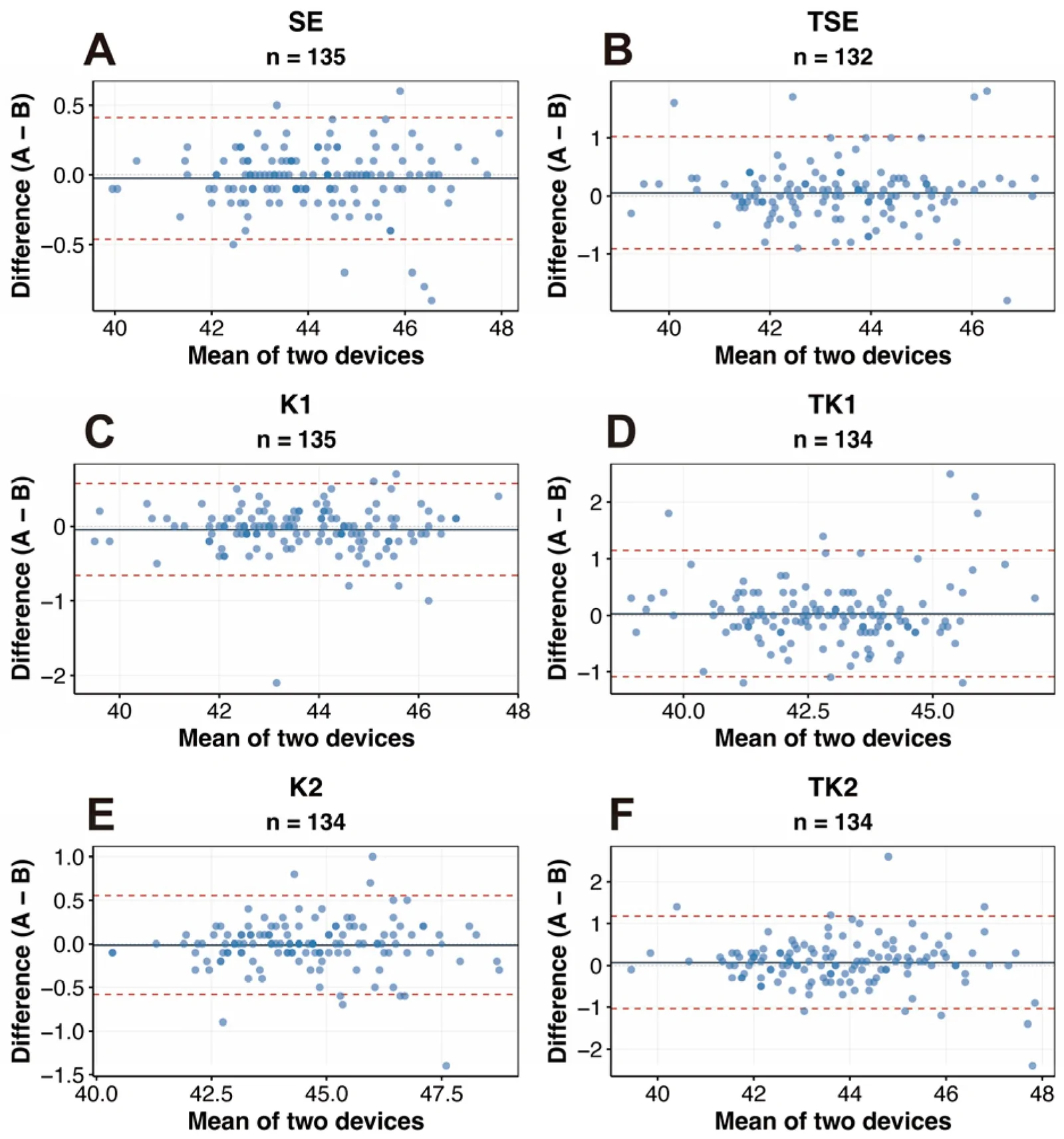
Bland–Altman analysis between Pentacam AXL WAVE and Pentacam. The inter-device consistency of SE (**A**), TSE (**B**), K1 (**C**), TK1 (**D**), K2 (**E**) and TK2 (**F**) is shown. The vertical axis represents the difference between the measurements of the two devices, whilst the horizontal axis represents the mean of the measurements from both devices. The grey solid line indicates the estimated bias, and the red dashed lines indicate the 95% limits of agreement.

Bland–Altman plots were further used to illustrate the distribution of interdevice differences and the 95% LoA for representative parameters (Figure 2 and Figure 3).

In Group 1, AL (Figure 2A) showed narrow LoA, indicating good agreement between Pentacam AXL WAVE and IOLMaster 700 for AL measurement; K1 (Figure 2C) showed a small mean difference and a relatively concentrated distribution of differences. The distributions of differences for ACD (Figure 2B) and K2 (Figure 2D) were wider than those for AL and K1, with relatively broad 95% LoA for K2. Diagnostic assessment for SE (Figure 2E) indicated heteroscedasticity, with the magnitude of interdevice differences varying across measurement levels. In contrast, WTW, total keratometry parameters, and some astigmatic vector components (Supplementary Figure S1) showed wider 95% LoA, suggesting limited clinical interchangeability. In Group 2, corneal curvature parameters (Figure 3) and anterior-surface astigmatic vector components showed relatively narrow LoA (Supplementary Figure S2). However, ACDint, Chord-related components, and total corneal astigmatic vector components showed greater variability (Supplementary Figures S3–S5). These findings suggest that certain extended anterior segment parameters and total corneal astigmatic vector components should not be used interchangeably across devices.

### 3.3. Sensitivity Analysis of Agreement Under Different Outlier-Handling Methods

**Figure 4:**
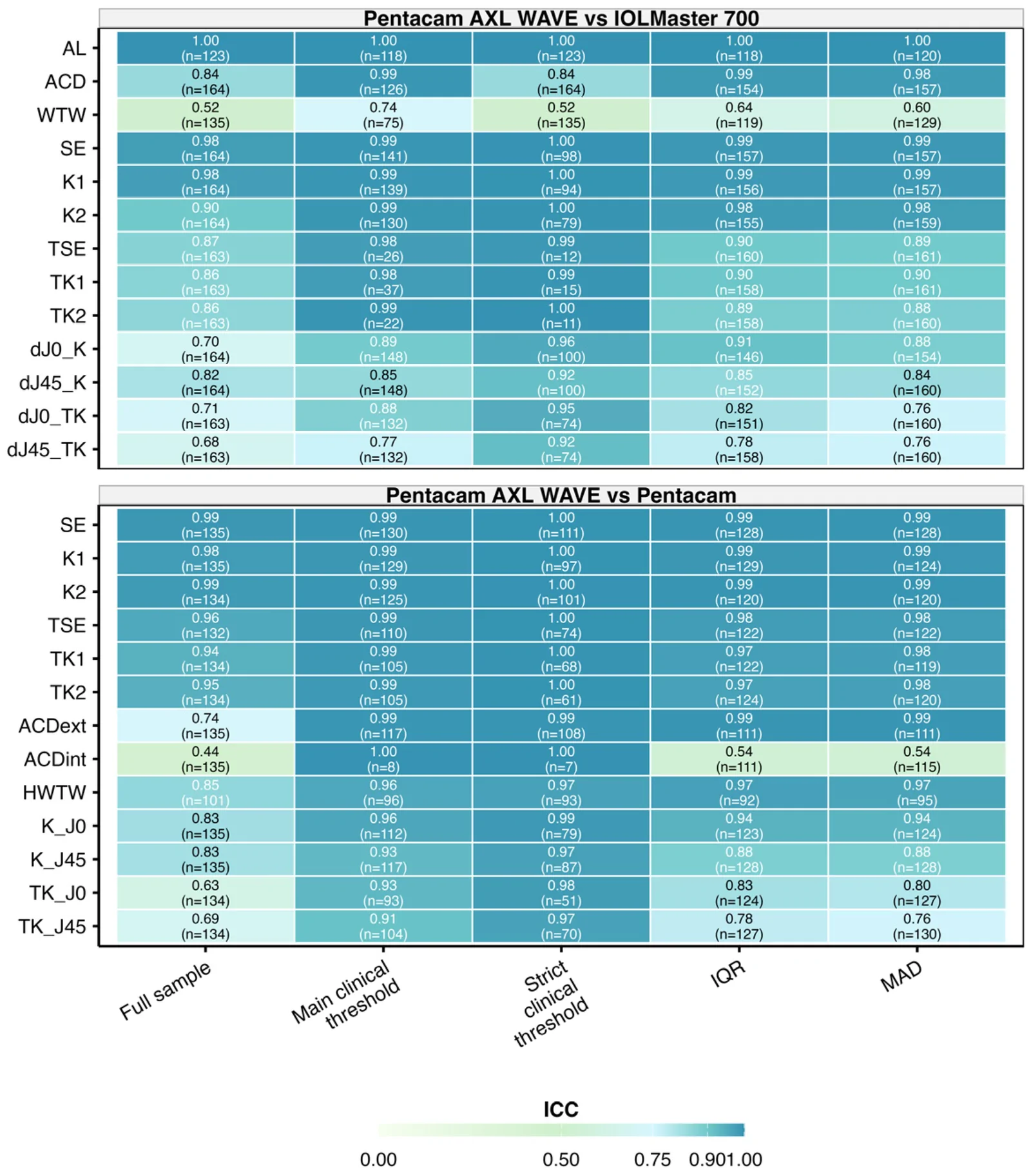
ICC heatmap across outlier-handling methods. The heatmap displays the ICC values and corresponding sample sizes for each parameter following the filtering methods: the full sample, filtering based on the primary clinical threshold, filtering based on a strict clinical threshold, filtering using the IQR method, and filtering using the MAD method. Darker colours indicate higher ICC values, and values where ICC > 0.85 are marked in white.

To evaluate the robustness of the agreement findings across different outlier-identification methods, ICCs were recalculated after applying the primary clinical threshold, the strict clinical threshold, the IQR method and the MAD method. Changes in ICC across outlier-handling methods are presented as a heatmap (Figure 4), and the complete CCC and Bland–Altman results are provided in Supplementary Tables S3 and S4.

The parameters showed heterogeneous responses to outlier-handling. Some core parameters maintained high ICCs across multiple methods, supporting robust agreement. In contrast, other parameters showed high ICCs only after a large number of discordant cases had been excluded using clinical thresholds, while ICCs decreased when most samples were retained using the IQR or MAD method. This pattern indicates that conclusions regarding interchangeability were method-dependent.

In the comparison between Pentacam AXL WAVE and IOLMaster 700, AL maintained an ICC approaching 1.00 across all methods, showing the highest stability. ACD, SE, K1, and K2 generally maintained high ICCs across different outlier-handling methods, though their 95% LoA should still be interpreted relative to predefined clinical thresholds. WTW continued to show only low-to-moderate agreement under the IQR and MAD methods, indicating that its interdevice differences were not mainly attributable to a few extreme outliers. TSE, TK1, and TK2 showed typical method-dependent patterns: ICCs were higher after application of the clinical-threshold method, but the corresponding sample size was markedly reduced; when most observations were retained using the IQR and MAD methods, ICCs decreased and the 95% LoA remained wide (Figure 4; Supplementary Table S3).

In the comparison between Pentacam AXL WAVE and Pentacam, SE, K1, K2, external anterior chamber depth (ACDext), and WTW showed relatively stable ICCs across outlier-handling methods, suggesting robust agreement between the two Scheimpflug-based devices for these parameters. ACDint showed clear sample-selection dependence, as its ICC approached 1.00 under the clinical-threshold method, yet the corresponding sample size was extremely small, and ICC decreased markedly when more samples were retained using the IQR and MAD methods. ACA180, COD, and some Chord components did not show consistently high agreement in the sensitivity analyses (Figure 4; Supplementary Table S4).

Astigmatic vector analysis further demonstrated the influence of outlier-handling methods on interdevice agreement. In Group 1, the ICC for dJ0_K, dJ45_K, dJ0_TK, and dJ45_TK increased under the clinical-threshold method but decreased to varying degrees under the IQR and MAD methods. This decline was more pronounced for total corneal astigmatic vector components. In Group 2, K_J0 and K_J45 generally performed better than TK_J0 and TK_J45 across different methods, and the latter showed a more pronounced decrease in ICC under the IQR and MAD methods. These results suggest that total corneal astigmatic vectors are more susceptible than anterior-surface astigmatic vectors to interdevice differences and to the choice of outlier definition (Figure 4; Supplementary Tables S3 and S4).

### 3.4. Analysis of Factors Associated with Interdevice Differences

**Figure 5:**
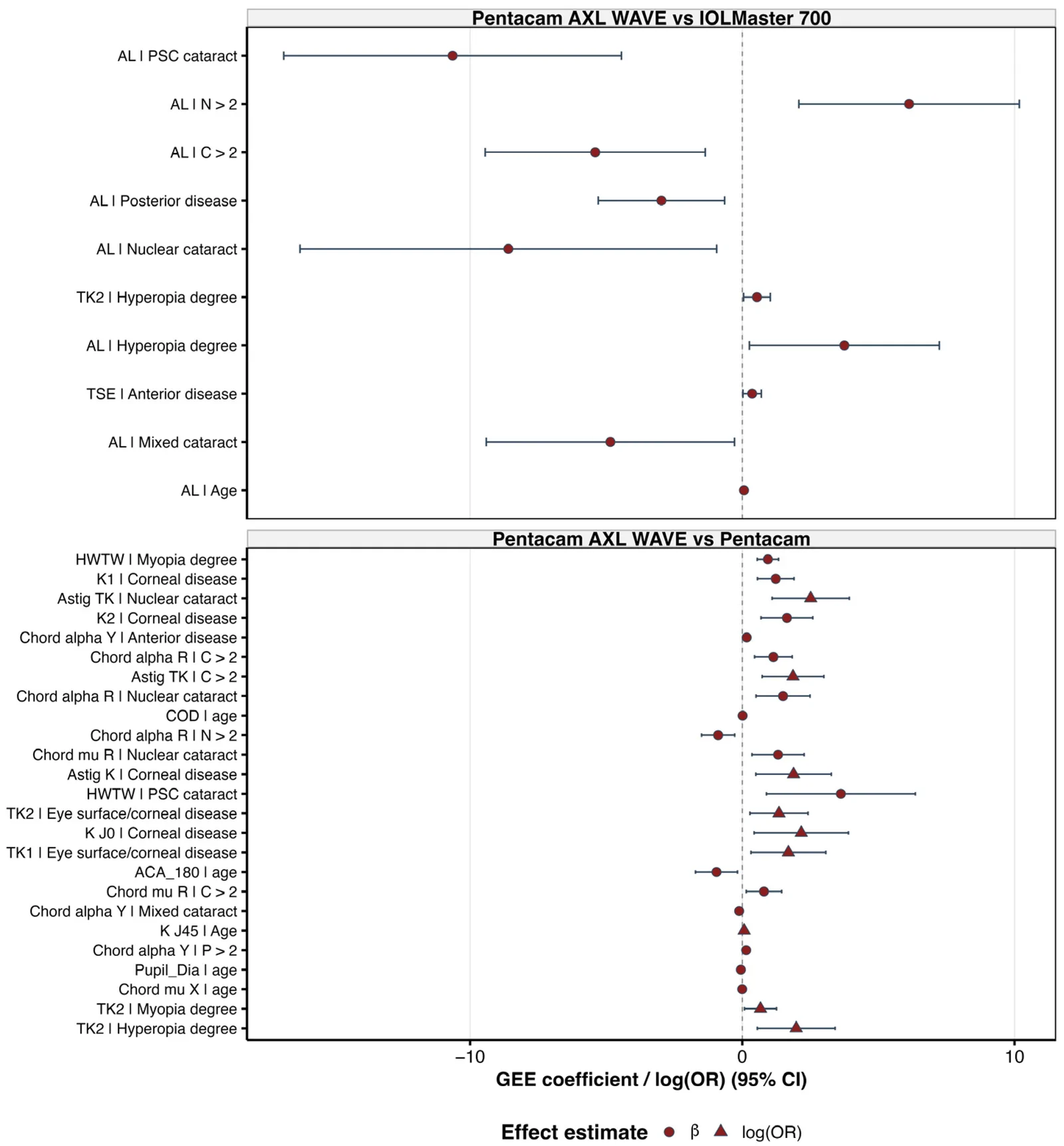
Clinical factors associated with inter-device measurement differences. The forest plot illustrates the key clinical factors associated with inter-device measurement differences in the GEE main model. Effect sizes are presented as regression coefficients, odds ratios or their transformed effect measures, along with 95% confidence intervals. In the forest plot, circles represent regression coefficients (β) for continuous outcomes, whereas triangles represent log-transformed odds ratios [log(OR)] for binary outcomes. The interpretation of effect sizes varies depending on the outcome type: for continuous difference outcomes, the effect size reflects the direction and magnitude of the inter-device difference; for binary outlier outcomes, the effect size reflects the relative likelihood of an outlier occurring at the clinical threshold.

To further investigate factors associated with interdevice differences, GEE models were constructed using interdevice difference metrics as outcome variables. Associations between clinical characteristics, including age, refractive status, cataract type, LOCS III grading, ocular comorbidities, and interdevice measurement differences were then evaluated. Based on the data characteristics of the different parameters, the outcome variables were defined as binary outliers, directional interdevice differences, or absolute interdevice differences. The main results with statistical significance, borderline significance, or potential clinical relevance are summarized in a forest plot (Figure 5), whereas the complete model results are presented in Supplementary Tables S5 and S6.

In comparing Pentacam AXL WAVE and IOLMaster 700, only a limited number of clinical factors were consistently associated with interdevice differences. Refractive status was associated with differences in total keratometry parameters, with the degree of hyperopia linked to increased interdevice differences in TK2. Anterior segment abnormalities were associated with interdevice differences in TSE and showed a borderline association with TK2. Although AL showed statistical associations with age, degree of hyperopia, cataract type, nuclear opacity grade, cortical opacity grade, and posterior segment-related abnormalities, AL also demonstrated an ICC close to 1.00 and narrow 95% LoA. Therefore, these associations likely reflect statistically detectable variation in very small measurement differences rather than clinically meaningful disagreement between devices. Overall, apart from total keratometry parameters, most clinical variables did not show broad or consistent associations with interdevice differences in Group 1 (Figure 5; Supplementary Table S5).

In the comparison between Pentacam AXL WAVE and Pentacam, factors associated with interdevice differences were mainly observed for corneal curvature, total corneal astigmatic vectors, Chord-related parameters, and selected extended anterior segment parameters. Ocular surface or corneal abnormalities were associated with interdevice differences in parameters such as K1, K2, TK1, TK2, and K_J0. Refractive status was also associated with several parameters. Specifically, the degree of myopia was associated with interdevice differences in HWTW and TK2, whereas the degree of hyperopia was associated with differences in TK2, ACDint, and Axial Sag BF Ratio. Cataract type and LOCS III grading were mainly associated with parameters such as total corneal astigmatic magnitude, TK_J0, Chord_alpha_r, Chord_alpha_y, Chord_mu_r, and ACA180. In addition, age was associated with parameters such as COD, ACA180, pupil diameter, Chord_mu_x, K_J45, and BAD-D, while posterior segment abnormalities were associated with interdevice differences in Chord_mu_r and ACDint (Figure 5; Supplementary Table S6).

Overall, factors associated with interdevice measurement differences varied considerably across parameters. In the comparison between Pentacam AXL WAVE and IOLMaster 700, clinical variables showed limited explanatory value for interdevice differences. In contrast, in the comparison between Pentacam AXL WAVE and Pentacam, ocular surface abnormalities, refractive status, cataract type, and LOCS III grading were associated with multiple corneal, total corneal astigmatic, and Chord-related parameters. These findings suggest that, even between devices based on the same Scheimpflug imaging technology, agreement for certain parameters may still be influenced by clinical phenotypes as well as differences in acquisition workflow, measurement-center localization, anterior and posterior surface reconstruction, and parameter-calculation algorithms.

## 4. Discussion

Using the Pentacam AXL WAVE as the primary comparison device, we evaluated its agreement and clinical interchangeability with IOLMaster 700 and Pentacam across preoperative biometric parameters used in cataract surgery. The findings showed that interdevice agreement was clearly parameter-dependent and context-dependent. Several core parameters showed good agreement, supporting their potential integration into routine preoperative assessment workflows. However, more complex parameters involving total corneal refractive power, astigmatic vectors, and extended anterior segment structures remained strongly device-dependent, indicating that cross-device substitution should be undertaken with caution. In addition, the associations between clinical phenotypes and interdevice differences varied across device comparisons, suggesting that interdevice disagreement may be influenced by both platform-related measurement differences and patient-specific ocular characteristics.

Among the evaluated parameters, AL and anterior corneal curvature measurements demonstrated the most consistent agreement. Pentacam AXL WAVE and IOLMaster 700 showed excellent agreement in AL measurements, with ICCs approaching 1.00 in both the full-sample analysis and the sensitivity analyses using different outlier-handling methods. Bland–Altman analysis further demonstrated narrow limits of agreement, supporting the robustness of this finding. Meanwhile, K1 and K2 showed good agreement in both comparisons involving Pentacam AXL WAVE, with more stable results observed in the comparison with Pentacam. As AL and anterior-surface K values are core parameters in routine IOL power calculation, these findings suggest that Pentacam AXL WAVE can provide clinically reliable measurements of both AL and anterior corneal curvature in routine cataract patients with acceptable image quality and regular corneal morphology, thereby helping to reduce repeated examinations and improve the efficiency of preoperative assessment.

In contrast to AL and anterior corneal curvature parameters, WTW showed limited agreement between Pentacam AXL WAVE and IOLMaster 700, and the agreement did not improve substantially after application of different outlier-handling methods. This finding suggests that interdevice differences in WTW were not attributable to a few extreme values, but instead reflected systematic differences in measurement definitions and image-recognition algorithms between the two device types. WTW measurement essentially depends on limbal boundary recognition, which represents a gradual corneoscleral transition rather than a discrete anatomical landmark, making its measurement susceptible to lighting conditions, iris pigmentation, palpebral fissure exposure, algorithmic recognition of the limbal transition zone, and device-specific boundary definitions [35]. Although WTW is not a key variable in most contemporary formulas for in-the-bag IOL power calculation, it is still used as an auxiliary biometric parameter in some formulas such as Barrett Universal II, Holladay 2, and VRF-G. WTW contributes to predicting effective lens position or IOL refractive power in their formulas [36,37]. Systematic measurement differences in WTW between devices may affect IOL refractive power calculation and final refractive outcome. In addition, WTW remains clinically significant for implants that depend on the transverse diameter of the anterior segment or for special surgical strategies, such as the size selection of phakic intraocular lenses, model evaluation of certain anterior chamber or iris claw intraocular lenses, and anterior segment space assessment for secondary intraocular lens implantation in uncapsulated eyes [38]. When WTW directly informs model- or size-related decisions, the device source should be kept consistent whenever possible, and cross-device mixing should be avoided.

Total keratometry parameters showed stronger device dependence. TSE, TK1, and TK2 showed relatively pronounced differences between Pentacam AXL WAVE and IOLMaster 700. Although ICCs increased markedly after application of the clinical-threshold exclusion method, the remaining sample size was substantially reduced. In contrast, when more observations were retained using the IQR and MAD methods, ICCs decreased and the LoA remained wide, suggesting unstable interchangeability. By comparison, TK1, TK2, and TSE showed high agreement between Pentacam AXL WAVE and Pentacam even in the full sample, with further improvement after the exclusion of clinical-threshold outliers. This difference may be related to device-specific differences in the acquisition of posterior corneal surface information and the calculation of total corneal refractive power. IOLMaster 700 derives TK parameters from an SS-OCT–based biometric platform and built-in algorithms, but Pentacam AXL WAVE and Pentacam reconstruct both the anterior and posterior corneal surfaces using Scheimpflug tomography. Nevertheless, total corneal curvature remains a composite parameter with substantial algorithmic dependence, and devices may still differ in reference axis, measurement zone, posterior surface modeling, and refractive power conversion. Therefore, in patients with previous corneal refractive surgery, abnormal corneal morphology, a high proportion of posterior corneal astigmatism, or a need for precise astigmatic correction, TK/TSE parameters obtained from different devices should not simply be regarded as fully equivalent data sources.

Astigmatic vector analysis provided additional support for the observed differences in total keratometry measurements. Astigmatism was decomposed into the orthogonal Jackson cross-cylinder vector components J0 and J45, thereby minimizing the influence of the cyclical nature of the astigmatic axis on conventional statistical analysis. The results showed that anterior-surface astigmatic vectors generally performed better than total corneal astigmatic vectors in both comparisons, whereas total corneal astigmatic vectors, particularly TK_J0 and TK_J45, showed greater instability under different outlier-handling strategies. This suggests that total corneal astigmatic vectors, as composite parameters integrating anterior and posterior corneal surface information and axis orientation, are more sensitive to device algorithms, posterior surface modeling, reference axes, and image quality. This finding is particularly important for toric IOL planning. The effectiveness of a toric IOL correction depends not only on astigmatic magnitude but also heavily on axis determination. Errors in axis measurement, differences in posterior corneal astigmatism estimation, or discrepancies in device reference axes may all contribute to postoperative residual astigmatism [39]. Therefore, when astigmatism results differ substantially across devices, toric IOL planning should incorporate a comprehensive assessment of multiple factors, including agreement between devices, corneal topographic characteristics, tear-film quality, the relative contribution of anterior and posterior corneal astigmatism, and measurement repeatability.

Beyond parameters used for routine IOL power calculation and astigmatic correction, extended anterior segment parameters and Chord-related indices should be considered auxiliary assessment metrics with greater device specificity. ACDint, ACA180, COD, and Chord α/μ-related components showed weaker agreement between Pentacam AXL WAVE and Pentacam, suggesting that these parameters depend more heavily on complex image boundary recognition, reference point definitions, and spatial geometric reconstruction, such that differences in device versions or algorithmic details may be amplified. Measurements of parameters such as Chord α/μ are influenced not only by the corneal vertex, pupil center, or visual-axis-related reference points, but also by pupil status, fixation quality, intraocular scatter, and image registration methods [40]. For multifocal and EDOF IOLs, Chord μ and related decentration parameters are commonly used to assess the potential risks of postoperative glare, halos, decentration sensitivity, and reduced visual quality [41,42]. If reference centers and acquisition conditions are not fully consistent across devices, cross-device substitution of these parameters may introduce bias in patient screening or IOL selection. Therefore, these parameters are more appropriately interpreted using the same device under similar examination conditions, and measurements from different devices should not be considered directly equivalent.

The sensitivity analyses provide important supplementary evidence for interpreting interdevice agreement. Previous agreement studies have often relied primarily on ICC, CCC, or Bland–Altman results, with relatively limited consideration of how different outlier-handling methods and post-exclusion sample sizes may affect conclusions regarding interchangeability. AL, SE, K1, and K2 maintained high agreement across multiple outlier-handling methods, supporting the robustness of these findings, whereas WTW, TK/TSE, total corneal astigmatic vectors, and ACDint showed clear method dependence. Notably, for some parameters, ICC increased markedly after outlier exclusion, accompanied by a substantial reduction in sample size. Such high agreement after exclusion is more likely to reflect agreement within a selected subset and cannot be directly extrapolated to general interchangeability in real-world clinical populations. Therefore, judgments regarding interdevice interchangeability should consider not only ICC/CCC, but also the Bland–Altman LoA, the proportion of outliers, and the representativeness of the post-exclusion sample.

Regression analyses were further used to explore the association between clinical phenotypes and interdevice differences, and the results suggest that interdevice disagreement may involve two distinct mechanisms. In the comparison between Pentacam AXL WAVE and IOLMaster 700, except for the associations of TK2 and TSE with a limited number of clinical phenotypes, most clinical variables did not show broad or stable associations with interdevice differences. This pattern suggests that these differences may primarily arise from the cross-platform measurement systems themselves, including differences in imaging principles, reference axes, acquisition pathways, limbal recognition, posterior corneal surface processing, and total corneal refractive power algorithms. Therefore, disagreement in WTW, TK/TSE, and total corneal astigmatism-related parameters is more likely to reflect differences in definitions or algorithms at the structural level, even in patients with relatively unremarkable clinical presentations. In contrast, in the comparison between Pentacam AXL WAVE and Pentacam, clinically related factors were more frequently associated with corneal curvature, total corneal astigmatism, Chord parameters, and selected extended anterior segment parameters. Because both devices are based on Scheimpflug imaging and have relatively high platform homology, inherent device differences are relatively reduced, making the influence of patient ocular status on measurement results more apparent. The associations of ocular surface or corneal abnormalities with differences in K values, TK values, and astigmatic vectors suggest that Scheimpflug measurements are sensitive to tear film stability, corneal regularity, and the quality of anterior and posterior corneal surface reconstruction. The associations of cataract type and LOCS III grading with Chord α/μ, total corneal astigmatism, and anterior chamber angle parameters suggest that lens opacity may amplify interdevice differences in complex anterior segment parameters by affecting fixation, pupil or corneal reflex localization, and image registration. Therefore, in cross-platform device comparisons, particular attention should be paid to structural disagreement caused by differences in definitions and algorithms. By contrast, in comparisons between Scheimpflug-based devices, greater attention should be given to the effects of ocular surface status, corneal morphology, lens opacity, and fixation quality on complex parameters.

These findings support a stratified application strategy for Pentacam AXL WAVE in preoperative cataract assessment. For patients undergoing routine monofocal IOL implantation with a stable ocular surface and regular corneal morphology, AL and anterior-surface K values obtained with Pentacam AXL WAVE can provide reliable preoperative information and may help streamline the examination workflow. For candidates for toric IOLs or other premium IOLs, which require greater precision in corneal curvature, astigmatic magnitude, and axis determination, preoperative biometry, IOL power calculation, and postoperative outcome interpretation should, whenever possible, be based on measurements obtained from the same device. Meanwhile, TK/TSE, astigmatic vectors, axis consistency, and corneal topographic morphology should be carefully reviewed to avoid refractive decision-making being affected by differences in total corneal astigmatism measurement. For candidates for multifocal or EDOF IOLs, Chord α/μ, pupil diameter, COD, and corneal morphological quality should be assessed using the same device under similar examination conditions and interpreted in the context of the patient’s visual needs and expectations. For patients with poor ocular surface status, abnormal corneal morphology, severe cataract opacity, or poor fixation, priority should be given to optimizing the ocular surface, repeating measurements, and reviewing the original images, rather than relying on a single device output for high-precision refractive decision-making.

This study has several strengths. Firstly, the study population was derived from a real-world cohort of patients undergoing preoperative cataract assessment, and overly restrictive exclusion criteria were not applied, enhancing the clinical relevance and generalizability of the findings. Secondly, in addition to routine biometric parameters such as AL, ACD, and keratometry values, the study included analyses of TK/TSE, astigmatic vector components, Chord-related parameters, and multiple extended anterior segment measurements, thereby aligning the evaluation of device interchangeability more closely with the practical needs of contemporary refractive cataract surgery. Thirdly, agreement was assessed using multiple complementary approaches, including ICC, CCC, and Bland–Altman analysis. Appropriate Bland–Altman methods were selected according to the characteristics of parameter differences, and several outlier-handling strategies were incorporated to reduce potential bias associated with reliance on a single statistical metric. Finally, GEE models were used to account for within-subject correlation arising from bilateral eye data, and the associations between clinical characteristics and interdevice differences were further explored, providing additional insights into the potential sources of disagreement for specific parameters.

Several limitations should also be acknowledged. Firstly, it is a single-center retrospective study, with the study population mainly drawn from our hospital’s preoperative cataract assessment cohort. Secondly, we used predefined clinical thresholds and various outlier handling strategies to assist in assessing inter-device interchangeability; however, for some extended parameters, there is currently a lack of universally accepted clinically acceptable ranges. Finally, this study primarily focuses on the inter-device consistency of preoperative measurement parameters, aiming to determine whether measurements obtained from different devices can be used for cross-reference. Future research should focus on prospective, multicenter studies including broader patient populations to validate the generalizability of these agreement findings. In addition, interdevice differences, within-device repeatability, and postoperative refractive and visual outcomes should be integrated to establish clinically acceptable thresholds for each parameter, thereby further clarifying the clinical value of Pentacam AXL WAVE in preoperative cataract assessment.

## 5. Conclusions

In summary, the agreement between Pentacam AXL WAVE and IOLMaster 700 or Pentacam should be interpreted at the parameter level rather than at the device level. Pentacam AXL WAVE showed high agreement with IOLMaster 700 for AL measurement and good comparability with Pentacam for anterior corneal curvature parameters such as K1, K2, and SE, supporting partial workflow integration in routine preoperative cataract assessment. In contrast, WTW, TK/TSE, total corneal astigmatic vectors, and certain extended anterior segment parameters showed varying degrees of interdevice disagreement. Therefore, Pentacam AXL WAVE should not be regarded simply as a complete substitute for IOLMaster 700 or Pentacam, but rather as a multimodal comprehensive assessment platform. When its parameter interchangeability boundaries are understood, Pentacam AXL WAVE can improve the efficiency of preoperative cataract evaluation and support individualized refractive decision-making.

## Figures and Tables

**Table 1:** Predefined clinical thresholds for outlier detection.

Parameter	Primary Threshold	Strict Threshold	Unit
AL	≥0.10	—	mm
ACD	≥0.10	—	mm
WTW/HWTW	≥0.50	—	mm
K1, K2	≥0.50	≥0.25	D
SE	≥0.50	≥0.25	D
TK1, TK2	≥0.50	≥0.25	D
TSE	≥0.50	≥0.25	D
K_J0, K_J45, TK_J0, TK_J45	≥0.25	≥0.125	D
ACA180, BAD-D, COD, pupil diameter, thinnest corneal thickness, and Chord α/μ X/Y components	—	—	Original units

## Data Availability

The datasets generated and analyzed during the current study are not publicly available due to patient privacy and institutional restrictions but are available from the corresponding author on reasonable request and with appropriate approval.

## Supplementary Materials

The following supporting information can be downloaded at https://www.mdpi.com/article/10.3390/jcm15176599/s1, Figure S1: Bland–Altman plots of corneal and WTW parameters; Figure S2: Bland–Altman plots of corneal parameters; Figure S3: Bland–Altman plots of anterior segment parameters; Figure S4: Bland–Altman plots of pachymetry and HWTW parameters; Figure S5: Bland–Altman plots of chord parameters; Tables S1: Pentacam AXL WAVE vs IOLMaster 700: Intraclass Correlation Coefficient (ICC), Concordance Correlation Coefficient (CCC) and Bland–Altman analysis; Table S2: Pentacam AXL vs Pentacam: Intraclass Correlation Coefficient (ICC), Concordance Correlation Coefficient (CCC) and Bland–Altman analysis; Table S3: Pentacam AXL WAVE vs. IOLMaster 700: Sensitivity Analysis; Table S4: Pentacam AXL WAVE vs. Pentacam: Sensitivity Analysis; Table S5: Pentacam AXL WAVE vs. IOLMaster 700: Analysis of Factors Associated with Variations Between Equipment; Table S6: Pentacam AXL WAVE vs Pentacam: Analysis of Factors Associated with Variations Between Equipment.
