# Supplementary material for: Clinical Interchangeability Boundaries of Pentacam AXL WAVE in Preoperative Biometry for Cataract Surgery: An Agreement Study with IOLMaster 700 and Pentacam
Source: J Clin Med. 2026 Aug 26;15(17):6599. doi: 10.3390/jcm15176599 (PMC13566189; doi:10.3390/jcm15176599)
Supplement: Supplementary file 1 [file jcm-15-06599-s001.zip › jcm-4457985-supplementary.pdf]

Supplementary Table S1 Pentacam AXL WAVE vs IOLMaster 700: Intraclass Correlation Coefficient (ICC), Concordance Correlation Coefficient (CCC) and Bland–Altman analysis

Parameters	Analysis of the full sample of raw data							Analysis following the removal of clinical outliers						
	n	ICC (95% CI)	P	CCC (95% CI)	Bias (95% CI)	95% LoA	LoA width	n	ICC (95% CI)	P	CCC (95% CI)	Bias (95% CI)	95% LoA	LoA width
**A. Standard biometric parameters**														
AL (mm)	123	0.9991 (0.9986, 0.9994)	<0.001	0.9991 (0.9987, 0.9994)	0.0337 (0.0158, 0.0517)	-0.1636 to 0.2310	0.3946	118	0.9998 (0.9992, 0.9999)	<0.001	0.9998 (0.9998, 0.9999)	0.0283 (0.022, 0.0346)	-0.0393 to 0.0959	0.1352
ACD (mm)	164	0.8434 (0.7669, 0.8922)	<0.001	0.8426 (0.7964, 0.8790)	-0.0891 (-0.1267, -0.0516)	-0.5665 to 0.3882	0.9547	126	0.9930 (0.9655, 0.9972)	<0.001	0.9930 (0.9902, 0.995)	-0.0324 (-0.0392, -0.0255)	-0.1086 to 0.0438	0.1524
WTW (mm)	135	0.5153 (-0.0859, 0.8145)	0.092	0.5135 (0.4327, 0.5861)	0.4711 (0.4296, 0.5126)	-0.0065 to 0.9487	0.9552	75	0.7360 (-0.0326, 0.9312)	0.062	0.7334 (0.6565, 0.7952)	0.3333 (0.3105, 0.3561)	0.1391 to 0.5276	0.3885
SE (D)	164	0.9793 (0.9718, 0.9848)	<0.001	0.9791 (0.9717, 0.9846)	0.0565 (-0.0017, 0.1148)	-0.6836 to 0.7967	1.4803	141	0.9928 (0.9888, 0.9952)	<0.001	0.9927 (0.9899, 0.9948)	0.0718 (0.0356, 0.1079)	-0.3539 to 0.4975	0.8514
K1 (D)	164	0.9763 (0.9679, 0.9825)	<0.001	0.9761 (0.9677, 0.9824)	0.0289 (-0.0340, 0.0918)	-0.7705 to 0.8283	1.5988	139	0.9930 (0.9901, 0.995)	<0.001	0.9929 (0.9901, 0.9949)	0.0443 (0.0067, 0.0819)	-0.3954 to 0.4841	0.8795
K2 (D)	164	0.8965 (0.8617, 0.9229)	<0.001	0.8959 (0.8611, 0.9224)	0.0295 (-0.1087, 0.1677)	-1.7268 to 1.7858	3.5126	130	0.9911 (0.9863, 0.9941)	<0.001	0.9910 (0.9874, 0.9936)	0.0778 (0.0353, 0.1204)	-0.4031 to 0.5588	0.9619
TSE (D)	163	0.8685 (0.1239, 0.9587)	0.012	0.8678 (0.8317, 0.8965)	0.8514 (0.7545, 0.9483)	-0.3763 to 2.0791	2.4554	26	0.9785 (0.8778, 0.993)	<0.001	0.9777 (0.9531, 0.9895)	0.1954 (0.1029, 0.2879)	-0.2534 to 0.6442	0.8976
TK1 (D)	163	0.8648 (0.2439, 0.9533)	0.006	0.8641 (0.8259, 0.8944)	0.8144 (0.7064, 0.9224)	-0.5542 to 2.1829	2.7371	37	0.9803 (0.9261, 0.9923)	<0.001	0.9797 (0.962, 0.9892)	0.1903 (0.1006, 0.2799)	-0.3366 to 0.7171	1.0537
TK2 (D)	163	0.8589 (0.1150, 0.9551)	0.013	0.8582 (0.8198, 0.8889)	0.9036 (0.7997, 1.0075)	-0.4133 to 2.2205	2.6338	22	0.9939 (0.9772, 0.9978)	<0.001	0.9936 (0.985, 0.9973)	0.1532 (0.0486, 0.2577)	-0.3090 to 0.6153	0.9243
**B. Astigmatism vector components**														
dJ0_K (D)	164	0.6974 (0.6095, 0.7684)	<0.001	0.6961 (0.6118, 0.7649)	-0.0149 (-0.0710, 0.0412)	-0.7277 to 0.6979	1.4256	148	0.8932 (0.8462, 0.925)	<0.001	0.8925 (0.8551, 0.9207)	-0.0524 (-0.0809, -0.024)	-0.3958 to 0.2910	0.6868
dJ45_K (D)	164	0.8176 (0.7497, 0.8669)	<0.001	0.8167 (0.7597, 0.8613)	0.0524 (0.0235, 0.0814)	-0.3156 to 0.4205	0.7361	148	0.8492 (0.775, 0.8968)	<0.001	0.8483 (0.7984, 0.8867)	0.0508 (0.0277, 0.0739)	-0.2282 to 0.3298	0.5580
dJ0_TK (D)	163	0.7147 (0.6307, 0.7823)	<0.001	0.7135 (0.6296, 0.7809)	-0.0243 (-0.0759, 0.0273)	-0.6779 to 0.6293	1.3072	132	0.8759 (0.8294, 0.9104)	<0.001	0.8751 (0.8302, 0.9087)	-0.0133 (-0.046, 0.0194)	-0.3854 to 0.3589	0.7443
dJ45_TK (D)	163	0.6795 (0.5762, 0.7595)	<0.001	0.6782 (0.5880, 0.7517)	0.0722 (0.0321, 0.1123)	-0.4361 to 0.5806	1.0167	132	0.7674 (0.6753, 0.8339)	<0.001	0.7660 (0.688, 0.8265)	0.0510 (0.0208, 0.0811)	-0.2926 to 0.3945	0.6871

The ICC was calculated using a two-factor mixed-effects model, absolute agreement, and a single measurement (ICC[A,1], following the Shrout & Fleiss / McGraw & Wong convention); the CCC was calculated as defined by Lin (1989). P-values were derived from significance tests for the ICC (H_0: ICC = 0). Interpretation criteria for ICC and CCC: ≥0.90 is excellent, 0.75–0.90 is good, 0.50–0.75 is moderate, and <0.50 is poor (Koo & Li, 2016). Bias = mean of the measurement differences between Device A and Device B; 95% CI is the confidence interval for bias; 95% LoA = bias ± 1.96 × SD (standard deviation of the differences); LoA width = Upper LoA − Lower LoA; a smaller value indicates better agreement. The post-exclusion column in Section A uses the clinical thresholds from the main analysis for each parameter; the ‘post-exclusion’ column for the astigmatism component in Section B uses the threshold |Δ| > 0.25 D (n = number of eyes remaining after exclusion).

Supplementary Table S2 Pentacam AXL vs Pentacam: Intraclass Correlation Coefficient (ICC), Concordance Correlation Coefficient (CCC) and Bland–Altman analysis

Parameters	n	Analysis of the full sample of raw data						Analysis following the removal of clinical outliers						
		ICC (95% CI)	P	CCC (95% CI)	Bias (95%CI)	95% LoA	LoA width	n	ICC (95% CI)	P	CCC (95% CI)	Bias (95%CI)	95% LoA	LoA width
A. Standard biometric parameters														
ACA_180 (°)	133	0.0726 (-0.0924, 0.2353)	0.195	0.0721 (-0.0893, 0.2299)	-6.7684 (-12.5483, -0.9885)	-72.8154 to 59.2785	132.0939	—	—	—	—	—	—	—
ACDext (mm)	135	0.7366 (0.6443, 0.8070)	<0.001	0.7352 (0.6540, 0.7997)	-0.0942 (-0.1625, -0.0259)	-0.8804 to 0.6920	1.5724	117	0.9927 (0.9866, 0.9957)	<0.001	0.9926 (0.9894, 0.9949)	-0.0217 (-0.0308, -0.0126)	-0.1192 to 0.0758	0.1950
ACDint (mm)	135	0.4425 (-0.0678, 0.7193)	0.052	0.4407 (0.3446, 0.5277)	-0.5844 (-0.6672, -0.5016)	-1.5375 to 0.3688	1.9063	8	0.9977 (0.9896, 0.9995)	<0.001	0.9974 (0.9890, 0.9994)	0.0175 (-0.0244, 0.0594)	-0.0806 to 0.1156	0.1963
Axial_Sag_BF_Ratio (ratio)	135	0.8176 (0.5634, 0.9071)	<0.001	0.8165 (0.7573, 0.8624)	0.0073 (0.0055, 0.0091)	-0.0137 to 0.0283	0.0420	91	0.9530 (0.7217, 0.9830)	<0.001	0.9525 (0.9313, 0.9673)	0.0043 (0.0034, 0.0053)	-0.0046 to 0.0132	0.0177
BAD_D (score)	135	0.7591 (0.6771, 0.8224)	<0.001	0.7577 (0.6824, 0.8171)	-0.0084 (-0.1227, 0.1058)	-1.3244 to 1.3075	2.6319	—	—	—	—	—	—	—
COD	135	0.1809 (-0.0612, 0.4917)	0.181	0.1798 (0.1287, 0.2299)	-0.0434 (-0.0463, -0.0404)	-0.0772 to -0.0095	0.0678	—	—	—	—	—	—	—
HWTW (mm)	101	0.8530 (0.7892, 0.8985)	<0.001	0.8517 (0.7889, 0.8969)	0.0396 (-0.0060, 0.0852)	-0.4134 to 0.4926	0.9060	96	0.9599 (0.9321, 0.9752)	<0.001	0.9595 (0.9407, 0.9724)	0.0427 (0.0198, 0.0656)	-0.1789 to 0.2644	0.4433
Pachy_Vertex_N (μm)	135	0.9186 (0.8068, 0.9577)	<0.001	0.9181 (0.8882, 0.9402)	-7.0667 (-9.0523, -5.0811)	-29.9293 to 15.7959	45.7252	99	0.9842 (0.8833, 0.9945)	<0.001	0.9840 (0.9769, 0.9889)	-4.2727 (-5.1425, -3.4030)	-12.8199 to 4.2745	17.0944
Pupil_Dia (mm)	71	0.5347 (0.3188, 0.6919)	<0.001	0.5312 (0.3554, 0.6707)	0.7220 (0.3168, 1.1271)	-2.6330 to 4.0770	6.7100	—	—	—	—	—	—	—
Thinnest (μm)	135	0.8047 (0.7032, 0.8687)	<0.001	0.8036 (0.7369, 0.8547)	-8.1926 (-11.8093, -4.5759)	-49.8363 to 33.4511	83.2873	—	—	—	—	—	—	—
K1 (D)	135	0.9803 (0.9724, 0.9859)	<0.001	0.9802 (0.9722, 0.9858)	-0.0430 (-0.0966, 0.0106)	-0.6602 to 0.5743	1.2346	129	0.9908 (0.9870, 0.9935)	<0.001	0.9907 (0.9869, 0.9934)	-0.0186 (-0.0562, 0.0190)	-0.4414 to 0.4042	0.8455
K2 (D)	134	0.9851 (0.9792, 0.9894)	<0.001	0.9850 (0.9790, 0.9893)	-0.0135 (-0.0630, 0.0360)	-0.5811 to 0.5540	1.1351	125	0.9932 (0.9904, 0.9953)	<0.001	0.9932 (0.9903, 0.9952)	0.0039 (-0.0306, 0.0384)	-0.3778 to 0.3857	0.7635
SE (D)	135	0.9902 (0.9862, 0.9930)	<0.001	0.9901 (0.9861, 0.9930)	-0.0244 (-0.0624, 0.0135)	-0.4617 to 0.4128	0.8745	130	0.9939 (0.9914, 0.9957)	<0.001	0.9938 (0.9913, 0.9956)	-0.0062 (-0.0365, 0.0242)	-0.3493 to 0.3370	0.6864
TK1 (D)	134	0.9421 (0.9195, 0.9585)	<0.001	0.9417 (0.9190, 0.9582)	0.0314 (-0.0662, 0.1291)	-1.0885 to 1.1514	2.2399	105	0.9877 (0.9819, 0.9916)	<0.001	0.9876 (0.9818, 0.9915)	-0.0078 (-0.0566, 0.0410)	-0.5022 to 0.4866	0.9889
TK2 (D)	134	0.9457 (0.9245, 0.9611)	<0.001	0.9453 (0.9241, 0.9608)	0.0694 (-0.0272, 0.1660)	-1.0391 to 1.1779	2.2171	105	0.9868 (0.9806, 0.9910)	<0.001	0.9867 (0.9805, 0.9909)	0.0419 (-0.0089, 0.0927)	-0.4729 to 0.5567	1.0297
TSE (D)	132	0.9564 (0.9391, 0.9689)	<0.001	0.9561 (0.9386, 0.9686)	0.0545 (-0.0304, 0.1395)	-0.9128 to 1.0219	1.9346	110	0.9897 (0.9851, 0.9929)	<0.001	0.9896 (0.9849, 0.9929)	0.0200 (-0.0253, 0.0653)	-0.4496 to 0.4896	0.9391
B. Astigmatism vector components														
K_J0 (D)	135	0.8300 (0.7692, 0.8759)	<0.001	0.8289 (0.7682, 0.8748)	0.0218 (-0.0254, 0.0689)	-0.5208 to 0.5643	1.0851	112	0.9626 (0.9461, 0.9741)	<0.001	0.9623 (0.9456, 0.9739)	-0.0037 (-0.0247, 0.0172)	-0.2230 to 0.2156	0.4386
K_J45 (D)	135	0.8263 (0.7642, 0.8732)	<0.001	0.8252 (0.7638, 0.8719)	-0.0259 (-0.0573, 0.0056)	-0.3882 to 0.3365	0.7247	117	0.9263 (0.8943, 0.9487)	<0.001	0.9257 (0.8947, 0.9478)	-0.0220 (-0.0412, -0.0028)	-0.2279 to 0.1839	0.4118
TK_J0 (D)	134	0.6310 (0.5168, 0.7231)	<0.001	0.6293 (0.5163, 0.7207)	0.0702 (0.0037, 0.1367)	-0.6930 to 0.8334	1.5264	93	0.9302 (0.8963, 0.9533)	<0.001	0.9295 (0.8963, 0.9524)	0.0258 (-0.0034, 0.0551)	-0.2524 to 0.3041	0.5565
TK_J45 (D)	134	0.6944 (0.5953, 0.7727)	<0.001	0.6928 (0.5945, 0.7707)	-0.0149 (-0.0579, 0.0281)	-0.5081 to 0.4784	0.9865	104	0.9110 (0.8715, 0.9388)	<0.001	0.9102 (0.8707, 0.9381)	-0.0057 (-0.0291, 0.0178)	-0.2421 to 0.2308	0.4728
C. Chord alpha / mu														
Chord_alpha_x (mm)	129	0.5145 (0.3758, 0.6306)	<0.001	0.5125 (0.3845, 0.6212)	-0.0464 (-0.1325, 0.0397)	-1.0149 to 0.9220	1.9369	—	—	—	—	—	—	—
Chord_alpha_y (mm)	129	0.4505 (0.3016, 0.5779)	<0.001	0.4485 (0.3205, 0.5605)	-0.0185 (-0.0649, 0.0280)	-0.5412 to 0.5043	1.0455	—	—	—	—	—	—	—
Chord_mu_x (mm)	125	0.7512 (0.6636, 0.8186)	<0.001	0.7497 (0.6617, 0.8174)	0.0134 (-0.0199, 0.0467)	-0.3549 to 0.3817	0.7367	—	—	—	—	—	—	—
Chord_mu_y (mm)	125	0.2356 (0.0626, 0.3947)	0.004	0.2342 (0.0666, 0.3889)	0.0180 (-0.0436, 0.0796)	-0.6639 to 0.6999	1.3638	—	—	—	—	—	—	—

The ICC was calculated using a two-factor mixed-effects model, absolute agreement, and a single measurement (ICC[A,1]); the CCC was calculated according to Lin's definition. P-values were derived from the significance test for the ICC (H_0: ICC = 0). Bias = the mean of the measurement differences between Device A and Device B; 95% LoA = bias ± 1.96 × SD.

Supplementary Table S3 Pentacam AXL WAVE vs IOLMaster 700: Sensitivity Analysis

Parameters	Main analysis (clinical threshold)				Strict clinical thresholds				IQR				MAD			
	n	ICC	CCC	95% LoA	n	ICC	CCC	95% LoA	n	ICC	CCC	95% LoA	n	ICC	CCC	95% LoA
**A. Standard biometric parameters**																
AL (mm)	118	0.9998	0.9998	-0.0393 to 0.0959	—	—	—	—	118	0.9998	0.9998	-0.0393 to 0.0959	120	0.9998	0.9998	-0.0538 to 0.1048
ACD (mm)	126	0.9930	0.9930	-0.1086 to 0.0438	—	—	—	—	154	0.9864	0.9863	-0.1468 to 0.0491	157	0.9845	0.9844	-0.1561 to 0.0529
WTW (mm)	75	0.7360	0.7334	0.1391 to 0.5276	—	—	—	—	119	0.6375	0.6355	0.1560 to 0.6591	129	0.6018	0.5999	0.1329 to 0.7337
SE (D)	141	0.9928	0.9927	-0.3539 to 0.4975	98	0.9974	0.9974	-0.2153 to 0.2924	157	0.9878	0.9878	-0.5007 to 0.6115	157	0.9878	0.9878	-0.5007 to 0.6115
K1 (D)	139	0.9930	0.9929	-0.3954 to 0.4841	94	0.9969	0.9969	-0.2350 to 0.2928	156	0.9874	0.9873	-0.5740 to 0.5912	157	0.9869	0.9868	-0.5794 to 0.6062
K2 (D)	130	0.9911	0.9910	-0.4031 to 0.5588	79	0.9964	0.9963	-0.2399 to 0.2948	155	0.9827	0.9826	-0.5869 to 0.7681	159	0.9792	0.9790	-0.6722 to 0.8237
TSE (D)	26	0.9785	0.9777	-0.2534 to 0.6442	12	0.9882	0.9872	-0.3502 to 0.3385	160	0.8975	0.8969	-0.1163 to 1.7138	161	0.8948	0.8942	-0.1259 to 1.7389
TK1 (D)	37	0.9803	0.9797	-0.3366 to 0.7171	15	0.9945	0.9941	-0.3024 to 0.2757	158	0.9038	0.9032	-0.2217 to 1.6955	161	0.8983	0.8977	-0.2541 to 1.7787
TK2 (D)	22	0.9939	0.9936	-0.3090 to 0.6153	11	0.9993	0.9992	-0.2665 to 0.2592	158	0.8859	0.8852	-0.0886 to 1.8275	160	0.8797	0.8790	-0.1086 to 1.8803
**B. Astigmatism vector components**																
dJ0_K (D)	124	0.9525	0.9521	−0.2719 to 0.1639	79	0.9819	0.9817	−0.1466 to 0.1061	146	0.9132	0.9126	−0.3753 to 0.2450	154	0.8847	0.8841	−0.4255 to 0.3222
dJ45_K (D)	135	0.8979	0.8972	−0.1781 to 0.2555	91	0.9594	0.9590	−0.1064 to 0.1433	152	0.8518	0.8510	−0.2089 to 0.3306	160	0.8386	0.8378	−0.2650 to 0.3669
dJ0_TK (D)	104	0.9427	0.9422	−0.2507 to 0.2402	63	0.9833	0.9831	−0.1284 to 0.1203	151	0.8233	0.8223	−0.4859 to 0.4477	160	0.7641	0.7630	−0.5885 to 0.5813
dJ45_TK (D)	116	0.8970	0.8962	−0.1848 to 0.2627	84	0.9400	0.9393	−0.1388 to 0.1616	158	0.7830	0.7819	−0.3479 to 0.4653	160	0.7601	0.7589	−0.3600 to 0.4931

Primary analysis: Data were excluded based on pre-specified clinical thresholds for each parameter (see Methods section). Astigmatism components were excluded using a stricter threshold of |Δ| > 0.125 D. IQR method: Tukey’s 1.5×IQR rule. MAD method: adjusted z-score > 3.5.

Supplementary Table S4 Pentacam AXL WAVE vs Pentacam：Sensitivity Analysis

Parameters	Main analysis (clinical threshold)				Strict clinical thresholds				IQR				MAD			
	n	ICC	CCC	95% LoA	n	ICC	CCC	95% LoA	n	ICC	CCC	95% LoA	n	ICC	CCC	95% LoA
A. Standard biometric parameters																
ACA_180 (°)	—	—	—	—	—	—	—	—	104	0.7679	0.7662	-14.3581 to 16.4370	108	0.7196	0.7178	-15.8654 to 18.7339
ACDext (mm)	117	0.9927	0.9926	-0.1192 to 0.0758	108	0.9948	0.9948	-0.0945 to 0.0397	111	0.9946	0.9946	-0.1014 to 0.0419	111	0.9946	0.9946	-0.1014 to 0.0419
ACDint (mm)	8	0.9977	0.9974	-0.0806 to 0.1156	7	0.9991	0.9990	-0.0663 to 0.0748	111	0.5443	0.5420	-0.6688 to -0.4598	115	0.5431	0.5409	-0.6867 to -0.4357
Axial_Sag_BF_Ratio (ratio)	91	0.9530	0.9525	-0.0046 to 0.0132	—	—	—	—	123	0.9019	0.9012	-0.0043 to 0.0183	124	0.9010	0.9003	-0.0048 to 0.0185
BAD_D (score)	—	—	—	—	—	—	—	—	124	0.8564	0.8554	-0.7361 to 0.7748	128	0.8272	0.8260	-0.8148 to 0.8561
COD	—	—	—	—	—	—	—	—	132	0.1208	0.1199	-0.0725 to -0.0135	132	0.1208	0.1199	-0.0725 to -0.0135
HWTW (mm)	96	0.9599	0.9595	-0.1789 to 0.2644	93	0.9718	0.9715	-0.1342 to 0.2245	92	0.9729	0.9726	-0.1252 to 0.2209	95	0.9667	0.9663	-0.1646 to 0.2404
Pachy_Vertex_N (μm)	99	0.9842	0.9840	-12.8199 to 4.2745	48	0.9947	0.9946	-6.4762 to 3.7679	129	0.9647	0.9644	-18.2290 to 4.5236	130	0.9647	0.9644	-18.4196 to 4.9735
Pupil_Dia (mm)	—	—	—	—	—	—	—	—	60	0.9371	0.9361	-0.5031 to 1.3748	60	0.9371	0.9361	-0.5031 to 1.3748
Thinnest (μm)	—	—	—	—	—	—	—	—	129	0.9620	0.9617	-19.6138 to 6.8386	129	0.9620	0.9617	-19.6138 to 6.8386
K1 (D)	129	0.9908	0.9907	-0.4414 to 0.4042	97	0.9969	0.9969	-0.2511 to 0.2346	129	0.9908	0.9907	-0.4414 to 0.4042	124	0.9923	0.9923	-0.4058 to 0.3590
K2 (D)	125	0.9932	0.9932	-0.3778 to 0.3857	101	0.9976	0.9976	-0.2186 to 0.2422	120	0.9948	0.9948	-0.3254 to 0.3419	120	0.9948	0.9948	-0.3254 to 0.3419
SE (D)	130	0.9939	0.9938	-0.3493 to 0.3370	111	0.9971	0.9971	-0.2384 to 0.2312	128	0.9946	0.9946	-0.3295 to 0.3170	128	0.9946	0.9946	-0.3295 to 0.3170
TK1 (D)	105	0.9877	0.9876	-0.5022 to 0.4866	68	0.9955	0.9954	-0.2870 to 0.2188	122	0.9747	0.9745	-0.7420 to 0.6750	119	0.9770	0.9768	-0.7002 to 0.6163
TK2 (D)	105	0.9868	0.9867	-0.4729 to 0.5567	61	0.9968	0.9968	-0.2233 to 0.2855	124	0.9724	0.9722	-0.6716 to 0.8135	120	0.9759	0.9757	-0.6069 to 0.7486
TSE (D)	110	0.9897	0.9896	-0.4496 to 0.4896	74	0.9968	0.9968	-0.2231 to 0.2825	122	0.9803	0.9801	-0.6414 to 0.6267	122	0.9803	0.9801	-0.6414 to 0.6267
B. Astigmatism vector components																
K_J0 (D)	112	0.9626	0.9623	-0.2230 to 0.2156	79	0.9869	0.9868	-0.1358 to 0.1334	123	0.9394	0.9390	-0.2747 to 0.2764	124	0.9369	0.9364	-0.2859 to 0.2811
K_J45 (D)	117	0.9263	0.9257	-0.2279 to 0.1839	87	0.9719	0.9716	-0.1415 to 0.1121	128	0.8811	0.8803	-0.2953 to 0.2281	128	0.8811	0.8803	-0.2953 to 0.2281
TK_J0 (D)	93	0.9302	0.9295	-0.2524 to 0.3041	51	0.9815	0.9811	-0.1377 to 0.1616	124	0.8264	0.8252	-0.4236 to 0.4914	127	0.8040	0.8027	-0.4811 to 0.5157
TK_J45 (D)	104	0.9110	0.9102	-0.2421 to 0.2308	70	0.9704	0.9700	-0.1212 to 0.1411	127	0.7844	0.7831	-0.3956 to 0.3466	130	0.7581	0.7567	-0.4273 to 0.3708
C. Chord alpha / mu																
Chord_alpha_x (mm)	—	—	—	—	—	—	—	—	112	0.9763	0.9761	-0.1834 to 0.1521	115	0.9716	0.9714	-0.2035 to 0.1598
Chord_alpha_y (mm)	—	—	—	—	—	—	—	—	118	0.7446	0.7430	-0.2333 to 0.2522	122	0.7068	0.7051	-0.2551 to 0.2745
Chord_mu_x (mm)	—	—	—	—	—	—	—	—	109	0.9289	0.9283	-0.1583 to 0.2009	111	0.9232	0.9225	-0.1712 to 0.2054
Chord_mu_y (mm)	—	—	—	—	—	—	—	—	108	0.6586	0.6565	-0.2796 to 0.3670	104	0.6984	0.6964	-0.2636 to 0.3214

The primary analysis uses the preset primary clinical threshold; the strict clinical threshold is a more stringent threshold; the IQR method refers to Tukey's 1.5×IQR rule; the MAD method refers to outlier identification based on the MAD. The table shows the remaining sample size (n), ICC, CCC and 95% LoA after excluding the corresponding outliers. For parameters without a pre-specified clinical threshold, the main analysis or strict clinical threshold columns are indicated by '—'.

Supplementary Table S5 Pentacam AXL WAVE vs IOLMaster 700：Analysis of Factors Associated with Variations Between Equipment

Parameters	Factors influencing	Effect estimate (β or OR, 95% CI)	Main analysis P-value	N
AL	Age	0.06 （0.00， 0.12）	0.038	45
AL	Degree of hyperopia	3.75 （0.26， 7.24）	0.035	45
AL	Nuclear cataract	-8.60 （-16.25， -0.94）	0.028	45
AL	Posterior subcapsular cataract	-10.64 （-16.85， -4.44）	<0.001	45
AL	mixed cataract	-4.85 （-9.41， -0.29）	0.037	45
AL	N>2	6.13 （2.08， 10.18）	0.003	45
AL	C>2	-5.40 （-9.45， -1.36）	0.009	45
AL	Posterior disease	-2.97 （-5.29， -0.65）	0.012	45
TK2	Degree of hyperopia	0.54 （0.05， 1.03）	0.032	61
TK2	Anterior disease	0.36 （-0.01， 0.74）	0.055	61
TSE	Degree of hyperopia	0.36 （-0.06， 0.78）	0.089	61
TSE	Anterior disease	0.36 （0.02， 0.70）	0.036	61

Depending on the type of outcome variable, odds ratios (OR) are reported for binary outcomes and beta (β) for continuous outcomes. Statistically significant results ($P < 0.05$) and borderline results ($0.05 \le P < 0.10$) are highlighted.

Supplementary Table S6 Pentacam AXL WAVE vs Pentacam：Analysis of Factors Associated with Variations Between Equipment

Parameters	Factors influencing	Effect estimate (β or OR, 95% CI)	Main analysis P-value	N
ACA_180	Age	β −0.95 (−1.72, −0.18)	0.016	118
ACA_180	Nuclear cataract	β −15.16 (−32.61, 2.28)	0.088	118
ACA_180	mixed cataract	β −17.98 (−35.27, −0.69)	0.042	118
ACDint	Degree of hyperopia	β 0.15 (0.01, 0.30)	0.041	120
ACDint	Nuclear cataract	β 0.26 (−0.04, 0.55)	0.086	120
ACDint	N>2	β −0.12 (−0.26, 0.02)	0.090	120
ACDint	Posterior disease	β 0.11 (0.00, 0.22)	0.050	120
Axial_Sag_BF_Ratio	Degree of myopia	OR 1.44 (0.94, 2.23)	0.098	120
Axial_Sag_BF_Ratio	Degree of hyperopia	OR 4.33 (1.14, 16.54)	0.032	120
Axial_Sag_BF_Ratio	Nuclear cataract	OR 4.56 (1.17, 17.83)	0.029	120
Axial_Sag_BF_Ratio	surface/corneal disease	OR 2.61 (0.94, 7.25)	0.065	120
Axial_Sag_BF_Ratio	Anterior disease	OR 0.25 (0.07, 0.88)	0.032	120
BAD_D	Age	β 0.03 (0.00, 0.05)	0.041	120
Chord_alpha_r	Nuclear cataract	β 1.50 (0.50, 2.49)	0.003	112
Chord_alpha_r	N>2	β −0.89 (−1.50, −0.28)	0.004	112
Chord_alpha_r	C>2	β 1.14 (0.45, 1.83)	0.001	112
Chord_alpha_x	surface/corneal disease	β 0.31 (−0.05, 0.67)	0.094	112
Chord_alpha_y	posterior subcapsular cataract	β −0.51 (−0.97, −0.06)	0.026	112
Chord_alpha_y	mixed cataract	β −0.12 (−0.21, −0.02)	0.018	112
Chord_alpha_y	P>2	β 0.14 (0.02, 0.26)	0.019	112
Chord_alpha_y	Anterior disease	β 0.16 (0.06, 0.26)	0.001	112
Chord_mu_r	Nuclear cataract	β 1.31 (0.35, 2.27)	0.007	109
Chord_mu_r	mixed cataract	β 0.75 (0.05, 1.46)	0.037	109
Chord_mu_r	C>2	β 0.80 (0.15, 1.45)	0.016	109
Chord_mu_r	surface/corneal disease	β 0.87 (0.10, 1.64)	0.027	109
Chord_mu_r	Posterior disease	β −0.60 (−1.19, −0.01)	0.046	109
Chord_mu_x	Age	β −0.00 (−0.01, −0.00)	0.019	109
Chord_mu_y	surface/corneal disease	β 0.19 (−0.03, 0.41)	0.089	109
Chord_mu_y	Posterior disease	β −0.10 (−0.23, 0.02)	0.094	109
COD	Age	β 0.01 (0.00, 0.02)	0.004	120
HWTW	posterior subcapsular cataract	β 3.62 (0.89, 6.36)	0.009	89
HWTW	Degree of myopia	β 0.94 (0.55, 1.33)	<0.001	89
K_J0	surface/corneal disease	OR 8.72 (1.54, 49.31)	0.014	120
K_J45	Age	OR 1.07 (1.01, 1.13)	0.018	120
K_J45	surface/corneal disease	OR 3.61 (0.94, 13.84)	0.061	120
K1	Degree of hyperopia	β 0.63 (−0.10, 1.37)	0.093	120
K1	surface/corneal disease	β 1.23 (0.56, 1.90)	<0.001	120
K2	surface/corneal disease	β 1.64 (0.69, 2.59)	<0.001	119
Pupil_Dia	Age	β −0.05 (−0.10, −0.01)	0.019	59
Thinnest	Anterior disease	β 0.90 (−0.13, 1.92)	0.088	120
Thinnest	Posterior disease	β −0.57 (−1.23, 0.09)	0.091	120
TK_J0	Age	OR 1.05 (0.99, 1.12)	0.083	119
TK_J0	Nuclear cataract	OR 5.56 (1.18, 26.19)	0.030	119
TK_J0	mixed cataract	OR 2.83 (0.85, 9.49)	0.092	119
TK_J0	C>2	OR 2.66 (0.95, 7.44)	0.063	119
TK_J0	surface/corneal disease	OR 3.15 (0.88, 11.26)	0.078	119
TK1	Degree of myopia	OR 1.67 (0.91, 3.04)	0.096	119
TK1	surface/corneal disease	OR 5.44 (1.38, 21.49)	0.016	119
TK2	Degree of myopia	OR 1.95 (1.08, 3.52)	0.026	119
TK2	Degree of hyperopia	OR 7.25 (1.74, 30.25)	0.007	119
TK2	surface/corneal disease	OR 3.85 (1.32, 11.18)	0.013	119

This table presents results from the main GEE model with $P < 0.05$ and borderline results with $0.05 \le P < 0.10$. Depending on the outcome type, odds ratios (ORs) are reported for binary outcomes and beta coefficients (β) are reported for continuous outcomes. n indicates the number of eyes included in the corresponding model.

Supplementary Figure S1 Bland–Altman plots of corneal and WTW parameters

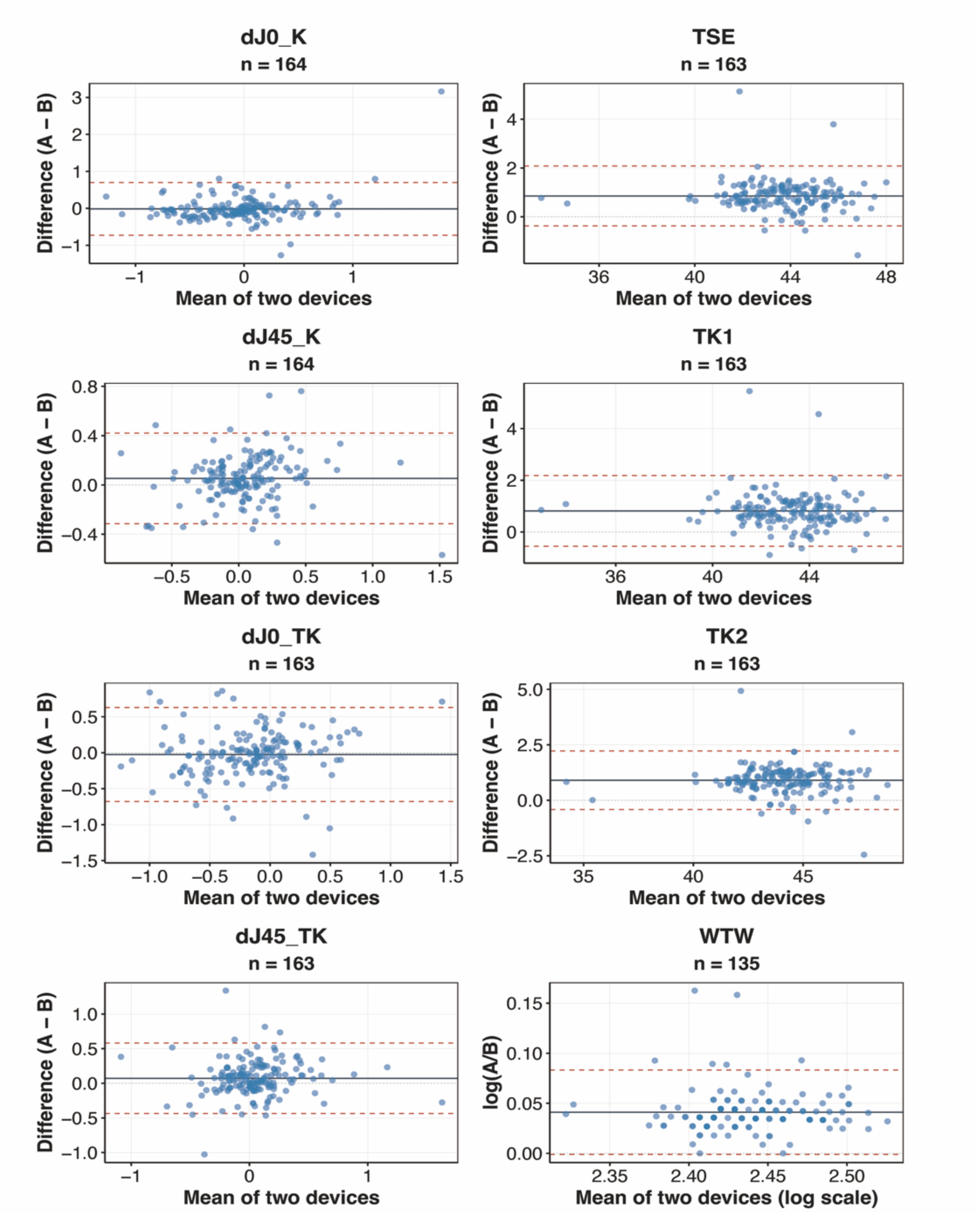

Bland–Altman plots of corneal-related and WTW parameters from the Pentacam AXL WAVE and the IOLMaster 700, including K_J0, K_J45, TK_J0, TK_J45, TSE, TK1, TK2 and WTW. The vertical axis represents the difference between the devices, and the horizontal axis represents the mean values for both devices; the blue line indicates the mean bias, and the red dotted line indicates the 95% limit of agreement.

Supplementary Figure S2. Bland–Altman plots of corneal parameters

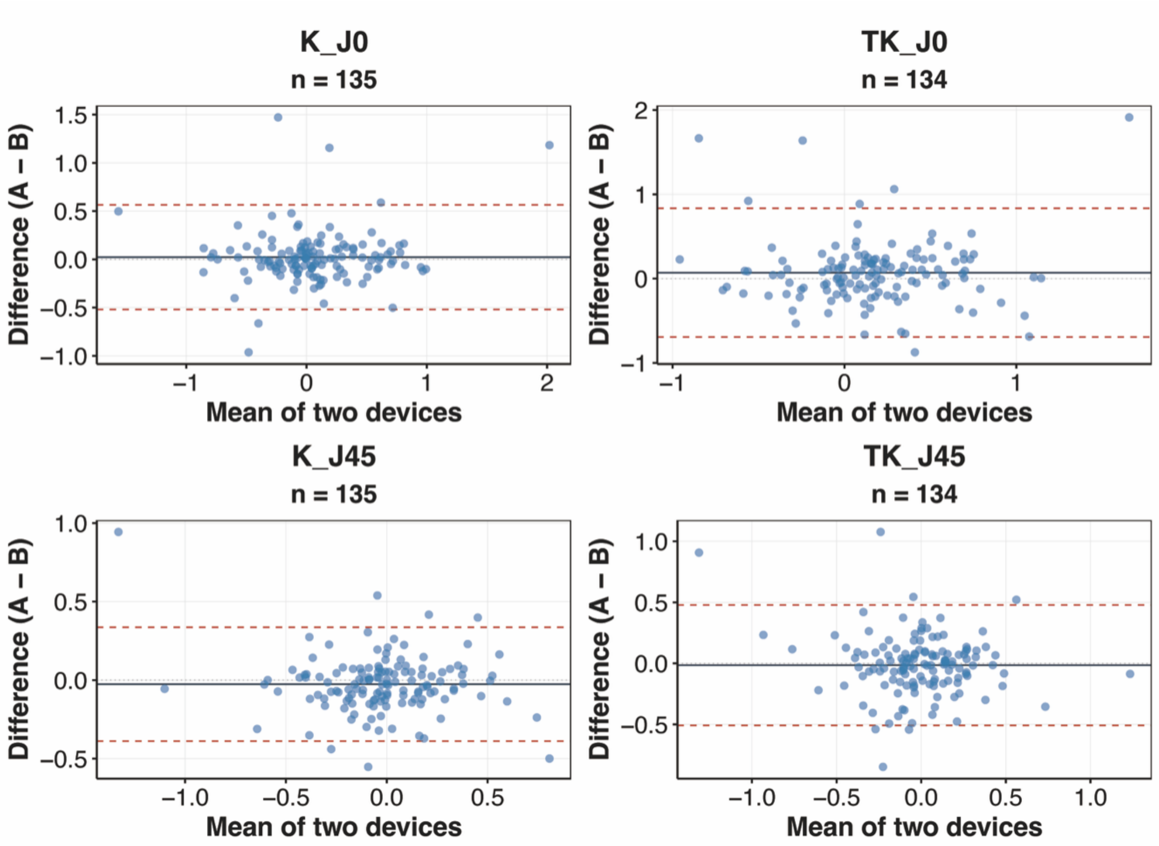

Bland–Altman plot comparing corneal parameters between the Pentacam AXL WAVE and the Pentacam, including K_J0, K_J45, TK_J0 and TK_J45. The vertical axis represents the difference between the two devices, and the horizontal axis represents the mean values for both devices; the blue line indicates the mean bias, and the red dotted line represents the 95% agreement limit.

Supplementary Figure S3 Bland–Altman plots of anterior segment parameters

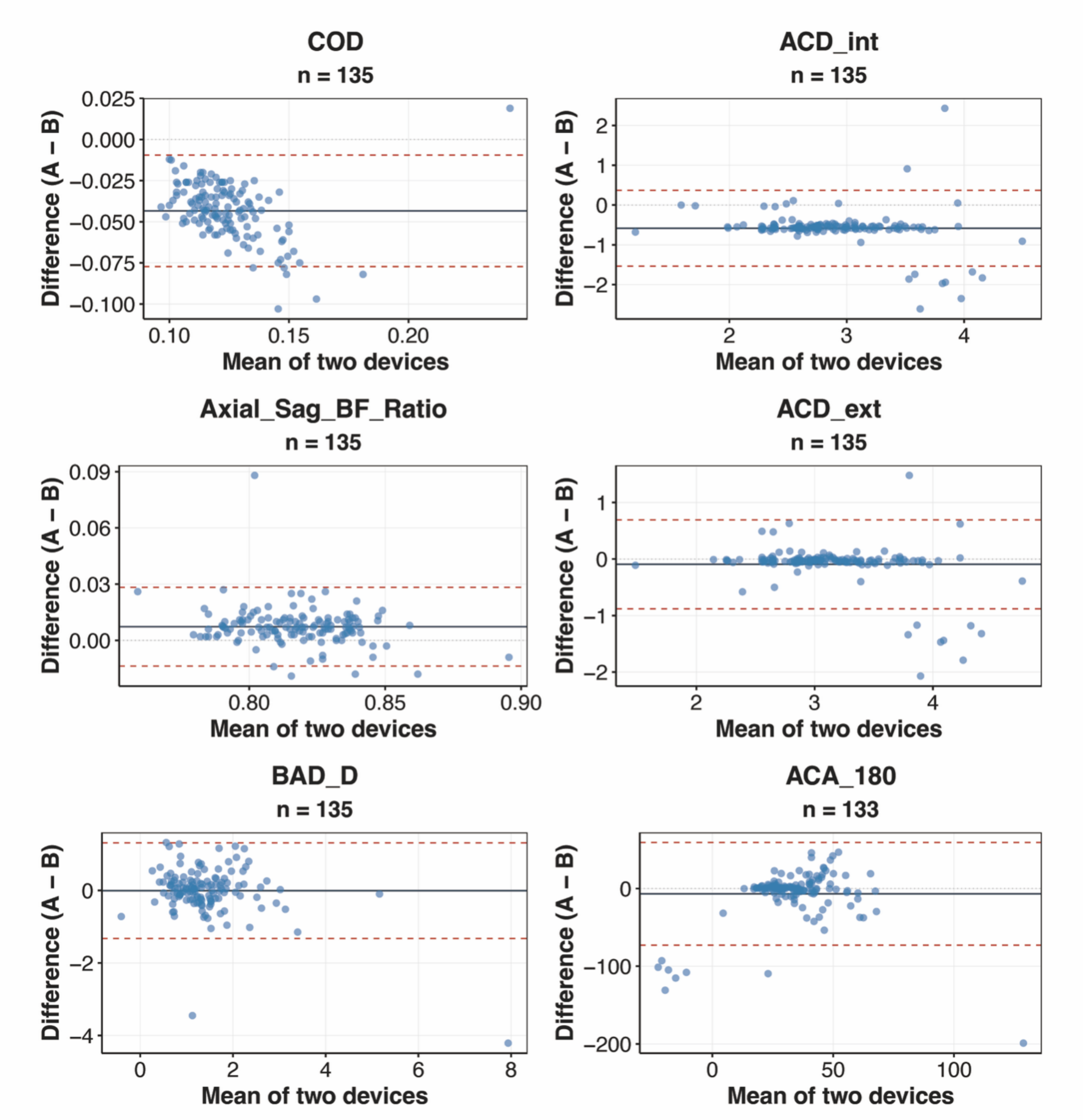

Bland–Altman plots comparing the Pentacam AXL WAVE with the previous generation Pentacam for relevant parameters, including COD, ACDint, ACDext, Axial_Sag_BF_Ratio, BAD_D and ACA_180. The vertical axis represents the inter-device difference, and the horizontal axis represents the mean values for both devices; the blue line indicates the mean bias, and the red dotted line represents the 95% limit of agreement.

Supplementary Figure S4. Bland–Altman plots of pachymetry and HWTW parameters

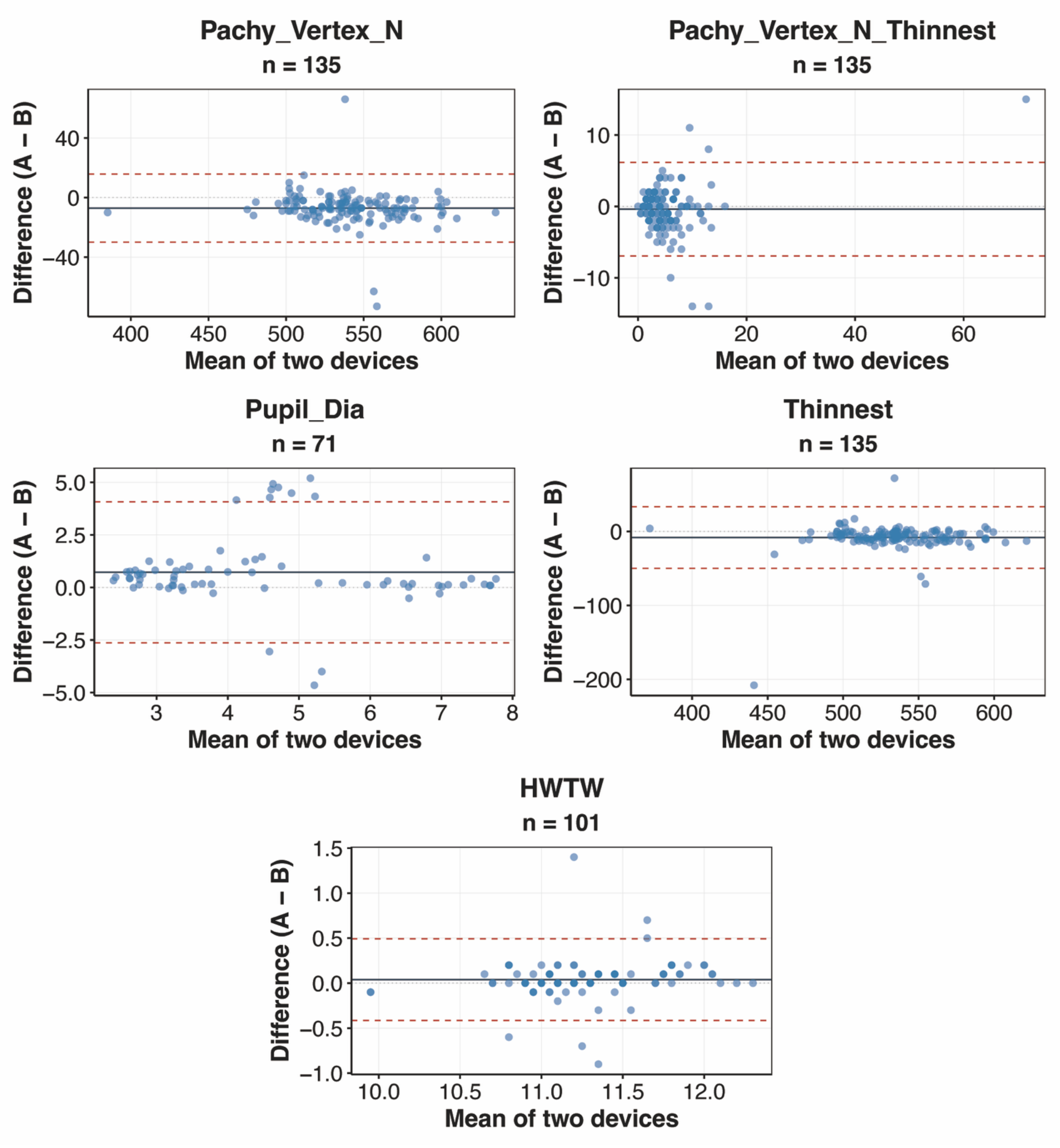

Bland–Altman plots comparing corneal thickness, pupil diameter and HWTW parameters between the Pentacam AXL WAVE and the Pentacam, including Pachy_Vertex_N, Pachy_Vertex_N_Thinnest, Thinnest, Pupil_Dia and HWTW. The vertical axis represents the difference between the devices, and the horizontal axis represents the mean values for both devices; the blue line indicates the mean bias, and the red dotted line represents the 95% limit of agreement.

Supplementary Figure S5. Bland–Altman plots of chord parameters

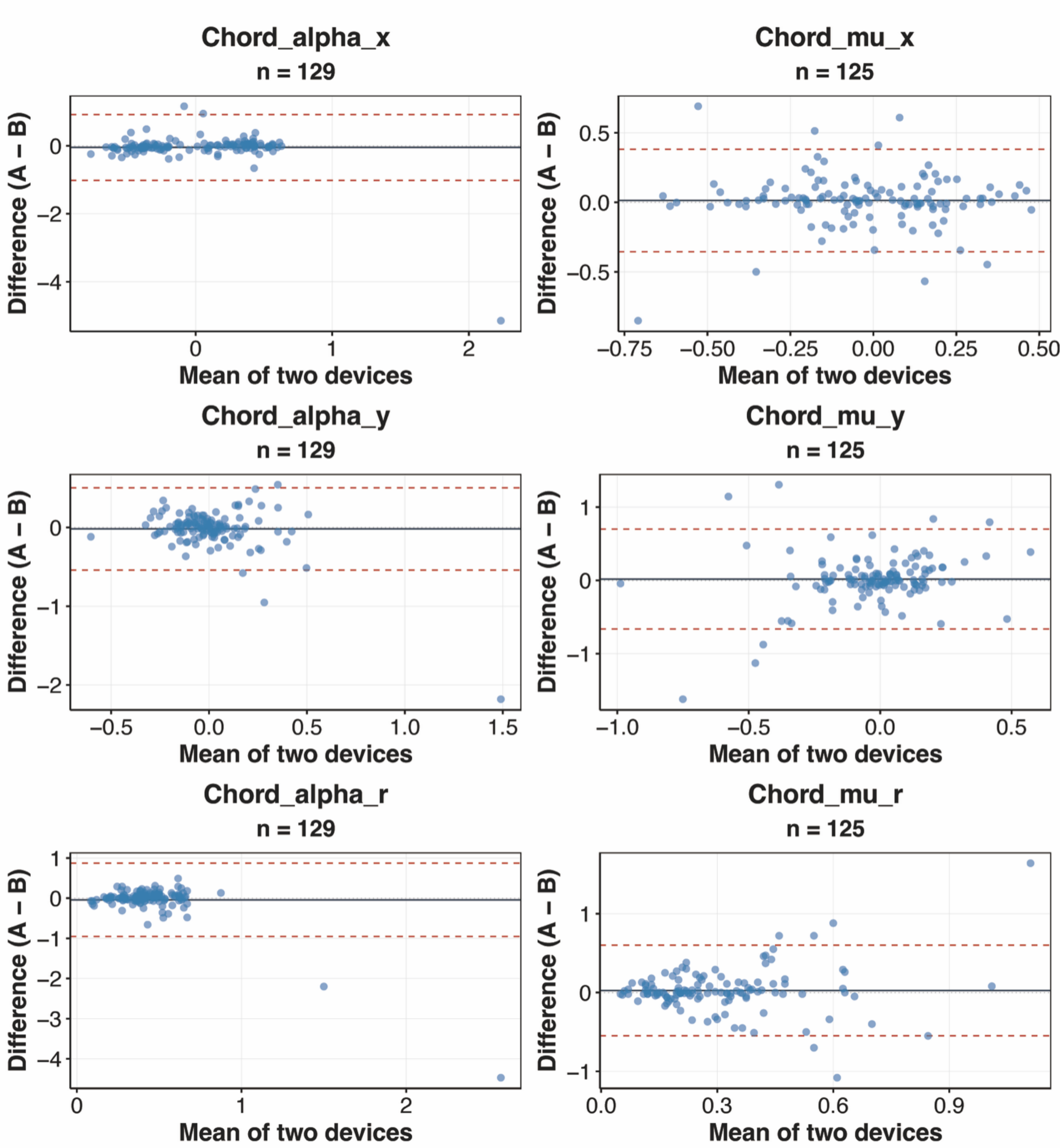

Bland–Altman plot of the α/μ parameters for the Pentacam AXL WAVE and Pentacam Chord, including Chord_alpha_x, Chord_alpha_y, Chord_alpha_r, Chord_mu_x, Chord_mu_y and Chord_mu_r. The vertical axis represents the difference between the devices, and the horizontal axis represents the mean values of both devices; the blue line indicates the mean bias, and the red dotted line indicates the 95% agreement limit.
